# Quantifying Site Heterogeneity in Microporous Aluminosilicates and Implications for Catalysis

**DOI:** 10.1021/acscatal.5c01948

**Published:** 2025-10-03

**Authors:** Edgard A. Lebrón-Rodríguez, Fillipp E. Salvador, Zahra Alikhani, Jerome M. Evans, Levi Callahan, Nicole K. Mitchell, Chenyao Huang, Sudipta Ganguly, Faysal Ibrahim, Ive Hermans

**Affiliations:** † Department of Chemical and Biological Engineering, 1466 University of Wisconsin-Madison, Madison, Wisconsin 53706, United States; ‡ Department of Chemistry, 5228 University of Wisconsin-Madison, Madison, Wisconsin 53706, United States; § The Wisconsin Energy Institute, University of Wisconsin−Madison, Madison, Wisconsin 53726, United States

**Keywords:** site heterogeneity, acid site titration, water effects, partially hydrolyzed, structure−performance relationships, alkane cracking, rate enhancement, zeolites

## Abstract

Zeolites and related microporous materials are key acid catalysts for many crucial transformations in both the gas and liquid phases for processes such as hydrocarbon refining, isomerization, and biomass upgrading. However, their catalytic behavior becomes complex under harsh hydrothermal conditions due to the formation of nonframework sites, which can significantly impact reaction rates and selectivity, complicating reproducibility and research evaluations. Therefore, in this work, we set out to establish characterization and titration protocols to identify and quantify site heterogeneity (i.e., differentiate between framework, partially hydrolyzed, and extraframework sites) of steamed microporous aluminosilicates, in contrast to solely using Brønsted and Lewis designations. For this purpose, we employ commercial MFI aluminosilicates (ZSM-5) of differing site heterogeneity and Si/Al ratios to quantify their site distribution through a combination of temperature-programmed desorption and FTIR protocols while contextualizing their effect on propane cracking rate constants. From the conclusions obtained, the present work provides a nuanced titration strategy on how to quantitatively determine the site heterogeneity of aluminosilicates and Al content without catalyst modification and with considerations for physisorbed species, base type, and size. We also reinforce literature observations of how water can induce changes in Al coordination even at ambient conditions, especially with increasing Al content, before catalysis, which adds variability in rate measurements. These observations and approaches should be extendable to other acidic zeolites and present ways to determine the site heterogeneity of materials in their dried state, in an accessible manner, that can serve as a starting point to evaluate structure–performance relationships.

## Graphical abstract

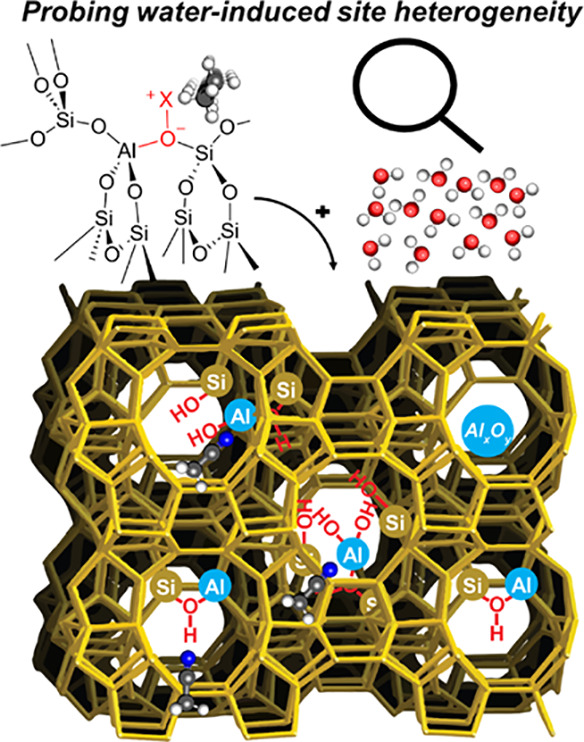

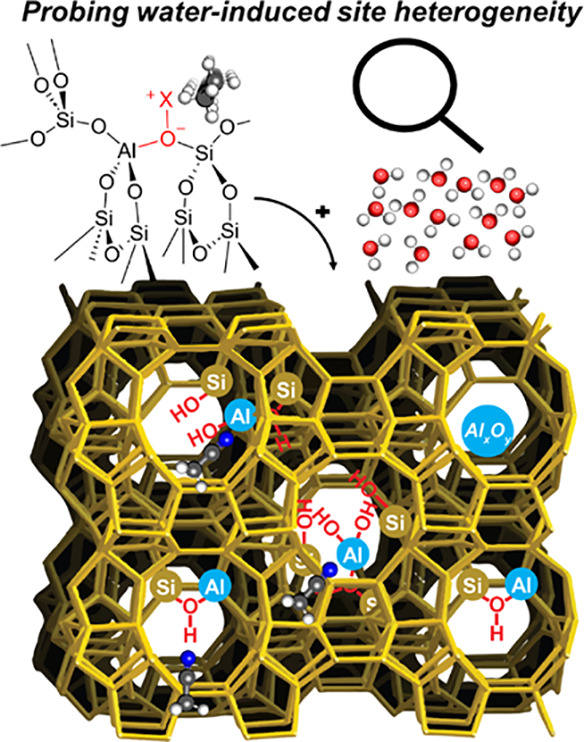

## 1 Introduction

Zeolites are important solid acids for catalytic transformations in traditional applications such as Fluid Catalytic Cracking (FCC) and emerging fields that include sugar/biomass upgrading and plastic chemical recycling.^1–4^ From the range of acid catalysts, zeolites are commonly viewed as defined materials, featuring well-defined repeating porous environments and SiOHAl (bridging hydroxyl) active sites within specific crystallographic tetrahedral (TO_4_) positions. However, when zeolites are exposed to regeneration conditions, steam formation causes catalyst structural changes, increasing the complexity of understanding the molecular-level details of reaction turnovers.^5–9^ This is because such conditions can lead to the creation of partially hydrolyzed (framework-associated) and extraframework sites, which affect reaction rates and selectivity in a nonlinear fashion. Additionally, variations in crystallization and calcination methods can significantly impact the formation of these sites at the synthesis stage, resulting in inconsistent rate measurements and varying selectivity patterns for “equivalent” catalysts, which complicates reproducibility.^10,11^ Therefore, shifting from a SiOHAl-centered site quantification to the determination of the whole acid site distribution (that includes partially hydrolyzed and extraframework species) is essential to describe and perform correlations with any acid–base reactions of interest catalyzed over solids.

Understanding the site heterogeneity (i.e., different distribution of framework, partially hydrolyzed, and extraframework sites) of zeolites allows the development of strategies to conserve carbon resources, especially nonrenewables. Petroleum has served as the carbon resource of choice to produce fuels and chemicals; however, waste streams in the form of CO_2_ and plastic solid waste, among others, have caused negative environmental consequences.^12,13^ As we have become more cognizant of our impact, more efficient utilization of our current carbon resources and integration of waste streams will be essential to shift to a more carbon-cognizant economy.^14,15^ The introduction of synthetic aluminosilicate zeolites in the 1960s revolutionized the FCC process by enhancing catalytic performance and selectivity to gasoline and minimizing coke deposition in contrast to amorphous silica–alumina.^1,16,17^ Apart from established effects of acid site density/proximity and porosity,^18,19^ zeolite active site speciation and distribution can affect conversion and selectivity significantly, of which the zeolite catalysis field has recently become increasingly cognizant.^20–25^ Now, the challenge is developing a catalytic formulation with the appropriate Al site type and distribution to activate inherently more nucleophilic double bonds (i.e., less acidic) present in larger quantities in alkene-rich and heavier petroleum residue while improving the stability of the current state-of-the-art catalysts.^1,2^ Therefore, zeolites will continue to play an important role as their shape selectivity through transition state stability and diffusion control will allow us to tune product selectivity to desired building blocks from heavier petroleum residue and more reactive plastic oil while minimizing coke deposition.

Seminal observations regarding the influence of active site speciation and distribution on hydrocarbon cracking rates were obtained by ExxonMobil in the 1980s.^26–28^ In 1986, specifically, they published a peer-reviewed article demonstrating that cracking rates could be increased in a nonlinear manner by steaming MFI zeolites, with the effect closely tied to the aluminum content.^26^ They attributed this rate enhancement to the formation of “enhanced acidic centers”, where the presence of paired aluminum framework atoms is an essential precursor for the creation of these active sites. Framework sites were quantified in pristine and steamed zeolites using the tetrahedral signal of ^27^Al solid-state NMR (ssNMR), cesium ion exchange titration, and metal content analysis after nonframework site extraction under reflux. Recently, Lercher and colleagues provided a compelling explanation for the origin of rate enhancement in hydrocarbon cracking. They observed that when SiOHAl acid sites are near Lewis acidic Al–OH groups—referred to as “extra lattice” aluminum in MFI—there is a significant increase in pentane cracking rates, with a 40-fold enhancement when normalized to SiOHAl–AlOH pairs.^5,6^ To establish a link between SiOHAl acid sites and the proximity of these sites, they used pyridine IR to examine the perturbation of the OH groups in Al–OH species. They proposed that the negative charge on the oxygen atom of the Al–OH group helps stabilize transition states by increasing activation entropy, which results in a later transition state that structurally and energetically resembles the products formed. Through continued investigation by various research groups, paired SiOHAl–SiOHAl also lead to higher cracking rates, where the literature has remained at the consensus that proximal sites lead to greater activation entropies.^5–9^ Synthetic advancements by Gounder and coworkers in understanding the hydrothermal synthesis of zeolites with organic and inorganic structure-directing agents (SDAs), along with their use of Co^2+^ ion-exchange titration to probe paired sites in MFI (as well as in CHA and MEL), have enabled the tuning and quantification of Al pairing and distribution in different void environments.^29–31^ Nonetheless, most evaluations on the effect of nonframework sites have been through comparison of catalysts with the presence and extraction of nonframework species. Typically, the (NH_4_)_2_SiF_6_ treatment is used to selectively remove Al–OH sites. In samples with Si/Al ratios of 25 or less, this treatment was found to considerably reduce the cracking rate, resulting in a linear correlation between pentane cracking rate and SiOHAl, similar to the unsteamed samples synthesized by ExxonMobil.^5,28^ However, a comprehensive acid site balance, one that accounts for the total aluminum content and distinguishes between nonframework, partially hydrolyzed, and extraframework sites, is still needed for rate correlations.

While the structure of SiOHAl sites is more defined, the same cannot be said for partially hydrolyzed and extraframework sites in aluminosilicate zeolites and their theoretical models. The uncertainty arises from the variety of potential sites, which is a result of aluminum’s inherent structural flexibility and variations in catalyst crystallization and calcination methods, leading to the formation of multiple species.^10,11,32,33^ Partially hydrolyzed aluminum sites, for example, are suggested to resemble species found in Sn-β, which are either singly or doubly hydroxylated [(SiO)_4 –__*n*_-Al­(OH)_*n*_]. This proposed structure is supported by comparisons of experimental and theoretical ^27^Al ssNMR data of chemical shift, quadrupolar coupling constant (*C*_Q_), and asymmetry parameter (η_Q_).^34–36^ However, there is no consensus on whether the bridging hydroxyl persists, despite its necessary role in balancing the framework hydrolysis reaction.^37^ Additionally, the positioning of hydroxyl groups in zeolites can influence their IR OH stretches depending on their chemical environment and hydrogen bonding. Sauer et al. demonstrated this by performing coupled-cluster-quality calculations on periodic models, which showed that the strength of internal H-bonds—and consequently the OH bond length—varies significantly depending on the framework position.^38^ As a result, accurately modeling the structure of all partially hydrolyzed sites remains a challenging task. In contrast, extraframework sites are thought to exist in various oxide and hydroxide forms, or as multinuclear clusters, but their exact structure is not well understood.^39,40^

Although modeling nonframework sites remains challenging, an important criterion for differentiating partially hydrolyzed sites from extraframework species lies in the reversible transition between octahedral and tetrahedral coordination during hydration and dehydration treatments.^35,41^ White et al. demonstrated, using quantitative ^27^Al ssNMR at various magnetic field strengths on dried H^+^-MFI-12.0 and H^+^-MFI-16.2 (both provided by Zeolyst), that no detectable hexacoordinated extraframework Al species were present before significant steam treatment (500 °C, 17 Torr of H_2_O).^35,36^ Upon exposure to water, hexacoordinated aluminum reappeared. However, the extent of catalyst hydration influenced the intensity of the extraframework signals, with the signal for partially hydrolyzed Al­(IV) sites either separating or converging with that of the framework Al­(IV). For hydrated samples, the signals of tetracoordinated partially hydrolyzed and framework Al sites overlapped. Therefore, framework and partially hydrolyzed sites should be distinguishable using titration methods when applied to commercial NH_4_^+^-MFI-12.0, H^+^-MFI-12.0, and H^+^-MFI-16.2 (Zeolyst). Only when the materials undergo more severe steaming are pentacoordinated and hexacoordinated aluminum species observed, providing a means to differentiate between partially coordinated and extraframework sites. However, the challenge persists in determining a suitable titration strategy to account for nonframework sites, as most studies that measure acid site counts in nonsteamed commercial MFIs report an acid/Al ratio of less than 1.

Therefore, in this work, we aim to characterize the full active site distribution of commercial MFI aluminosilicates (ZSM-5) with varying site heterogeneity and Si/Al ratios. Our goal is to quantify their site distributions in an accessible manner using a combination of temperature-programmed desorption and FTIR protocols while correlating these distributions with propane cracking rates. Through a quantitative assessment of NH_4_^+^-MFI-11.4, an in situ activated catalyst used directly prior to any reaction or analysis, we show that it contains only framework sites and is insensitive to the proton affinity and size of the titrant base. In contrast, H^+^-MFI-11.4, an ex situ activated catalyst stored under ambient laboratory conditions (25 °C, RH 20–50%), undergoes ambient-induced hydrolysis of SiOHAl sites to Al–OH species. This transformation leads to greater variation in Brønsted acid site (BAS) and Lewis acid site (LAS) counts. Nevertheless, total acid densities could be determined with ethylamine and deuterated acetonitrile. However, when generalizing site titrations to other Si/Al ratios of commercial Zeolyst samples (nominal Si/Al of 140, 40, 25, and 15), ethylamine has its pitfalls with temperature-programmed desorption (TPD) measurements that involve wet purge steps that are ineffective with low proton density samples and can cause changes in site coordination at higher temperatures. In contrast, with deuterated acetonitrile (CD_3_CN), the lack of extensive purge protocols and the CN group can capture the distribution of sites in a zeolite (without obfuscation of physisorbed species) as partially hydrolyzed sites form a Lewis adduct and framework sites hydrogen bond to acetonitrile instead of through Brønsted acid–base interaction that can be influenced by van der Waals interactions and proximity. Under-quantification with wet purges and variation in BAS and LAS counts is exemplified with titration measurements on γ-Al_2_O_3_. Propane cracking rate constant measurements when normalized by bridging hydroxyl counts determined with CD_3_CN provide reasoning for the enhancement and decrease in rate that is ascribed to the formation of nonframework sites and loss of SiOHAl, respectively. Overall, Brønsted acid site counts do not vary with the selected base for MFI materials considered in this study with Si/Al ≥ 15; however, variation was observed for high aluminum content (Si/Al of 11.5), steamed materials, or specific framework types. All things considered, the present work provides a nuanced titration strategy on how to quantitatively determine the site heterogeneity of steamed aluminosilicates with differing site distributions (i.e., framework, partially hydrolyzed, and extraframework sites) and Al content without catalyst modification and with considerations of physisorbed species, base type, and size. We also reinforce literature observations of how water can induce changes in Al coordination even at ambient conditions, especially in high Al-containing materials, before catalysis. These changes contribute to variability in rate measurements alongside treatment effects in the presence of water and deviations in titration counts. The strategies and observations outlined here are extendable to other acidic zeolites and offer an accessible means to evaluate site heterogeneity in dried-state materials, providing a foundation for studying structure–performance relationships.

## 2 Experimental Methods

### 2.1 Materials and Ion Exchange

#### 2.1.1 Commercial MFI Zeolites and NH_4_^+^ Form Activation

Zeolitic materials with different Si/Al (11.5 to 140) ratios were obtained from commercial vendors Zeolyst, Tosoh, and ACS Material in either the H^+^ or NH_4_^+^ form (Si/Al measured with ICP-OES). Zeolyst: NH_4_^+^-MFI-12.0 (CBV 2314), NH_4_^+^-MFI-16.2 (CBV 3024E), NH_4_^+^-MFI-27.8 (CBV 5524G), NH_4_^+^-MFI-40.4 (CBV 8014E), NH_4_^+^-MFI-147 (CBV 28014), NH_4_^+^-BEA-12.8 (CP814E*), and H^+^-FAU-16.6 (CBV 720); Tosoh provided by the IZA Catalysis Commission: H^+^-MFI-12.2 and NH_4_^+^-MFI-11.4; ACS Material: NH_4_^+^-CHA-11.7. It is important to note that materials received in the H^+^-form were not calcined and used as is. Once materials are in the H^+^-form, they are stored at laboratory ambient conditions of 25 °C and relative humidity (RH) between 20 and 50%. Standard *ex situ* activation of NH_4_^+^ form zeolite to its H^+^ form (i.e., H^+^-MFI-12.0) was performed by preparing loosely packed thin beds of materials in quartz boats and subsequently treating them in a muffle furnace (Yamato FO300CR) with flowing dry air (Drierite 26800-dried house air, 150 mL min^–1^ g_cat_^–1^) at 500 °C (1 °C min^–1^) for 4 h.^42,43^ Nonetheless, when not enough material was utilized, a minimum air flow rate of 2 L min^–1^ was used as a lower limit of air residence time (∼4 min) inside the treatment chamber. In addition, all materials activated in a muffle furnace were ground into a fine powder with an agate mortar before being placed in quartz boats. To produce H^+^-MFI-12.0 materials with varying site heterogeneity (i.e., to differentiate between framework, partially hydrolyzed, and extraframework sites), the treatment temperature was varied from 600 to 800 °C (in 100 °C increments; 10 °C min^–1^) with a longer 20 h hold.^44^

#### 2.1.2 Na^+^ and NH_4_^+^ Ion Exchange

Zeolites were converted to the Na^+^ form through aqueous-phase ion exchange with a 1 M NaNO_3_ solution (1 M NaNO_3_, >98%, Sigma-Aldrich), using 150 mL g_cat_^–1^ while stirring at ambient conditions (ca. 25 °C) for 24 h in a closed PFA container (Savillex). To ensure complete ion exchange at room temperature, the procedure was performed three times (in total). For comparison, ion exchange was also performed at 80 °C (temperature of liquid) with glassware under reflux conditions with 1 M NaNO_3_ solution (1 M NaNO_3_, >98%, Sigma-Aldrich) or 1 M NH_4_NO_3_ solution (1 M NH_4_NO_3_, >98%, Sigma-Aldrich). Afterward, solids were recovered and washed with deionized water (400 mL g_cat_^–1^ in total) through centrifugation. Na^+^ and NH_4_^+^-form catalysts were then dried overnight (>8 h) at 100 °C and at room temperature under vacuum (Edwards RV8, 0.1 Torr) to remove physisorbed water, respectively. It is important to dry NH_4_^+^-form zeolites in this manner as heating causes NH_3_ desorption. Exchanged Na^+^ form zeolites were then pretreated by preparing thin beds of materials in quartz boats and subsequently treating them in a muffle furnace (Yamato FO300CR) with flowing dry air (150 mL min^–1^ g_cat_^–1^; Drierite 26800 dried house air) at 550 °C (2 °C min^–1^) for 6 h.

### 2.2 Material Characterization

#### 2.2.1 Powder X-ray Diffraction (PXRD)

Prior to analysis, materials were ground into a fine powder by using an agate mortar and then placed onto backloading sample holders to minimize the preferential alignment of crystal orientations. Powder XRD patterns were recorded using a Bruker D8 Advance Diffractometer with Cu–K_α_ radiation (λ = 1.5406 Å, 40 kV, 40 mA), which was collimated with a 0.6 mm slit. The diffractometer was equipped with a Lynxeye detector and a 0.2 mm nickel (Ni) foil to filter out K_β_ radiation. A beam knife was positioned just above the sample to reduce air scattering at low angles without obstructing the diffracted X-rays. Diffraction patterns were collected over a 2θ range of 5–50°, with a step size of 0.02° and an exposure time of 1 s per step. The diffraction patterns presented here were recorded without background subtraction or smoothing. The experimental conditions for acquiring the diffraction patterns were based on guidelines from the IZA Synthesis Commission.^45^

#### 2.2.2 Ar Adsorption Isotherm

Approximately 0.0900–0.1100 g of the sample (ground into a fine powder) was accurately weighed into a check-sealed tube (without a fill rod) and then evacuated under dynamic vacuum using a Micromeritics VacPrep 061 unit, achieving a pressure of less than 0.1 Torr. The degassing procedure for the argon adsorption isotherms involved heating the sample to 120 °C for 1 h, followed by heating to 350 °C overnight (>8 h) with no ramp rate (as the VacPrep 061 model lacks this feature). After the degassing step, the samples (sealed with check seals) were reweighed and then immediately attached to a Micromeritics 3-Flex instrument. Prior to analysis, the samples were vacuumed for an additional 1 h to reach a pressure of approximately 10^–5^ Torr. Argon adsorption–desorption isotherms were measured at −186 °C (87.5 K), with the saturation pressure recorded at each data point. After the isotherm was completed, the samples were vacuumed again for 1 h at 25 °C to ensure a pressure of about 10^–5^ Torr and to measure the analysis and ambient space with helium. To prevent potential helium trapping in micropores, the empty space was measured after the analysis.^46^

For data analysis, the micropore volumes were determined by identifying the minimum on a semilogarithmic plot of ∂(V_ads_)/∂(ln­(*P/P*_*o*_)) versus ln­(*P/P*_*o*_), where the first peak corresponds to the micropore filling transition and the subsequent minimum marks the end of the micropore filling. Mesopore volumes were determined by measuring the total volume at *P/P*_*o*_ = 0.96 and subtracting the micropore volume contribution. Micropore size distributions were calculated using an H^+^ or metal-form zeolite cylindrical pore model based on nonlocal density functional theory (NLDFT) in the 3-Flex software. To ensure accurate micropore analysis and prevent errors at low pressures, thermal transpiration corrections were applied.^47^ The Barrett–Joyner–Halenda (BJH) model was used to calculate pore size distributions from the adsorption branch, incorporating the Harkins–Jura thickness curve and the Faas correction.^48^

#### 2.2.3 Elemental Analysis (ICP-OES)

Samples were prepared in triplicate by dissolving 0.0180–0.0220 g of solid material in 1.50 mL of hydrofluoric acid (HF, Sigma-Aldrich, ACS reagent, ≥48%) and allowing them to digest overnight (>12 h). Following digestion, the samples were diluted with 40 ± 0.05 mL of a 6 wt % HNO_3_ solution in Milli-Q water (prepared from 70 wt % HNO_3_, Sigma-Aldrich, ACS reagent), reducing the concentration of HF to 2 wt %. To prevent the formation of solids in the nebulizer during analysis and avoid excessive sample dilution, the samples were not complexed with H_3_BO_3_.^49^ Calibration standards for each metal were prepared by serial dilution of 1000 ppm of ICP standards (Sigma-Aldrich, TraceCERT, ±4 ppm) using 6 wt % HNO_3_ in deionized water. The elemental compositions of the samples were determined by using inductively coupled plasma-optical emission spectroscopy (ICP-OES) on an Agilent 5110 ICP-OES system equipped with an HF introduction apparatus. Prior to the measurement, the instrument was calibrated for each element. During analysis, sample measurements were corrected for drift and viscosity differences using 10 ppm of indium (In) as an internal standard.^50^ Absorbance readings were collected through the axial detector at wavelengths of 396.2, 288.2, 589.6, and 325.6 nm for Al, Si, Na, and In, respectively.

#### 2.2.4 NH_3_ and Alkylamine Temperature Programed Desorption (TPD)

NH_3_ and alkylamine TPD experiments were performed with either dry or wet purge steps that remove physisorbed base and hydrogen-bonded structures that allow to determine acid site densities as rigorously described in the literature.^42,43,51–53^ Zeolites in their NH_4_^+^ or H^+^ form (0.0290–0.0310 g) were sieved to 180–250 μm and weighed in a U-tube quartz reactor supported over a quartz wool plug. The sample was then attached to a Micromeritics Autochem II 2920 Chemisorption Analyzer equipped with a MKS Cirrus 2 mass spectrometer (MS) to quantify desorbed gaseous titrants evolved from catalysts through calibrated NH_3_ (16 *m*/*z*) and ethylamine (30 *m*/*z*) signals (no calibration cylinders for other alkylamines). In particular, NH_4_^+^-form TPD experiments are useful to determine the number of ion-exchangeable sites. For this method, samples were equilibrated under He (Airgas UHP He, 50 sccm) for 30 min at 40 °C (quartz-sheathed thermocouple) with subsequent desorption as denoted below for gas phase saturated samples. In terms of samples that require gas saturation, prior to acid site measurements, the materials were treated at 500 °C (10 °C min^–1^) for 1 h under Ar flow (Airgas UHP Ar, 50 sccm). After thermal pretreatment, the sample was cooled at a 20 °C min^–1^ rate to 160 or 150 °C and thereafter saturated with the desired base, where for lower-temperature adsorption experiments (<150 °C), the sample was exposed to the base for 5 min at this temperature before continuing to cool down under base flow to 40, 100, 112, or 137 °C. For alkylamine titration experiments, samples were further saturated in a flowing stream comprising of either ethylamine (Airgas EA 1000 ppm in balance UHP He, 50 sccm) or Ar (Airgas UHP Ar, 50 sccm) vapor-saturated alkylamine (reservoir at 25 °C filled with *n*-propylamine or *iso*-propylamine; Sigma-Aldrich, ≥98%) at 40, 100, 112, 137, or 150 °C for 2 h. Physisorbed species were purged in flowing He (Airgas UHP Ar, 50 sccm) either dry for 2.5 h or wet (reservoir at 25 °C filled with MQ water) for 8 h at the desired temperature. For NH_3_ titration experiments, samples were further saturated in flowing gaseous NH_3_ (Airgas 1000 ppm in balance UHP He, 50 sccm) for 2 h and then purged either dry or wet in a similar fashion at 40 or 160 °C. After titrant saturation and purge treatments, TPD was performed in flowing He (Airgas UHP He, 50 sccm; appropriate contact time to completely desorb the base) to 600 °C (10 °C min^–1^) with a 30 min hold, during which the U-tube reactor effluent was sent to the MS via heated lines/sections held at 150 °C. After each TPD experiment, NH_3_ (16 *m*/*z*) and ethylamine (30 *m*/*z*) signals were calibrated with mixtures in a balance of He (Airgas UHP He) to avoid any influence of MS signal drift.

#### 2.2.5 In Situ Transmission IR

Deuterated acetonitrile (CD_3_CN, Sigma-Aldrich, >99.9%, 99.96 atom % D) and pyridine (Py; C_5_H_5_N, Acros Organics, 99.5%) adsorption experiments were conducted following similar procedures as described by Wichterlová and Thibault-Starzyk et al., respectively.^54,55^ Before the analysis, CD_3_CN was purified using four freeze–pump–thaw cycles (with isopropanol and dry ice) via the IR vacuum system (Figure S1; Pfeiffer Vacuum HiCUBE with LN_2_ trap, MKS 925 MicroPirani, 1 × 10^–4^ Torr) and was then stored in a miniature sample cylinder (Swagelok SS-4CS-TW) with a closed needle valve (Swagelok Nupro BK Gas Shut-Off Valve). Meanwhile, Py was dried over molecular sieves (3A, Sigma-Aldrich, 1.6 mm pellets; used with a 4.4 g of 3A/L ratio) and stored in a valve-sealed bubbler. For sample preparation, ambient-stored zeolites (0.015–0.020 g, powder) were homogenized and compressed (3.5 tons for 2 min) into a self-supporting wafer (1 cm^2^) using an automated press (Pyke AutoCrush IR). For NH_4_^+^-form samples, the pressing pressure was kept at ≤2 tons to prevent incomplete NH_3_ desorption. The *in situ* IR cell, containing the ambient zeolite sample, was connected to a manifold with a flow and vacuum system (Pfeiffer Vacuum HiCUBE with LN_2_ trap, MKS 925 MicroPirani, 1 × 10^–4^ Torr) and coupled to a Bruker Vertex 70 spectrometer equipped with a liquid nitrogen-cooled Mercury–Cadmium–Telluride (MCT) detector. Typically, 128 scans with a resolution of 4 cm^–1^ were averaged to produce a spectrum in the 4,000 to 400 cm^–1^ range. Spectra were recorded relative to an empty cell with the reference background taken (256 scans) under dynamic vacuum (1 × 10^–4^ Torr) at the appropriate temperature (25 or 150 °C) for the OH region, CD_3_CN, and Py adsorption studies. The *in situ* SS transmission IR cell (Figure S2) included (1) a stainless steel body with ZnSe windows on the sides and a separate two-channel stainless steel attachment for holding the sample connected to the flanged top of the cell; (2) two resistive heating rods (Tutco CH26625, 300 W) in series with PID control (Love Controls 16B); and (3) a K-type thermocouple (Omega) positioned 1 mm from the sample. A custom stainless-steel manifold was used for sample pretreatment under gas flow (Alicat MFC, 50 sccm) or vacuum and to dose-controlled amounts of gaseous titrants into the cell.

For CD_3_CN and Py experiments, samples were activated in the *in situ* cell under air (5A and Drierite 27068 L68GP dried house air, Alicat MFC, 50 sccm g_cat_^–1^) at 500 °C (10 °C min^–1^) for 1 h. After thermal pretreatment, the gas flow was switched to N_2_ (5A and Drierite 27068 L68GP dried house N_2_, Alicat MFC, 50 sccm g_cat_^–1^), and the sample was cooled at 20 °C/min to 150 °C. At this temperature, the zeolite was saturated with Py (Py; C_5_H_5_N, Acros Organics, 99.5%) in flow (Alicat MFC, N_2_ = 50 sccm) for 30 min or evacuated until the pressure reached 1 × 10^–4^ Torr (Pfeiffer vacuum HiCUBE with LN_2_ trap, MKS 925 MicroPirani) to further cool the sample to room temperature (25 °C) and perform CD_3_CN dosing. For CD_3_CN experiments, the sample was isolated from the vacuum system, and doses of CD_3_CN (approximately 2 × 10^–7^ mol) were introduced into the cell, allowing each dose to equilibrate for 3 to 10 min (indicated by stable spectral features) before collecting a final IR spectrum. Continued dosing occurred until the sample became saturated (cell pressure ∼1 Torr and 2265 cm^–1^ for gas-phase CD_3_CN). Physisorbed CD_3_CN and Py molecules were removed by evacuation at 25 or 150 °C for 1 h, respectively, or until bridging hydroxyl and Al–OH peaks remained unchanged. For data processing, spectra were normalized to the middle Si–O–Si overtone of the zeolite framework (ca.1937–1788 cm^–1^) and baseline-corrected using OPUS software, followed by subtraction of the spectrum of the parent zeolite. Peak deconvolution of the CD_3_CN IR peaks into individual Gaussian components (2330–2310, 2300–2297, and fixed 2285, 2275, and 2265 cm^–1^) was performed with Origin software using fitting constraints from the literature.^56^ It should be noted that peak deconvolution of H^+^-MFI-12.0 (Figure S3) without including Al–OH species (2285 cm^–1^) resulted in an overestimation of SiOHAl and acid/Al ratios >1, unlike counts obtained using other bases. A more accurate estimate of SiOHAl counts was achieved by including Al–OH species (2285 cm^–1^) for all samples exhibiting Al–OH bands (3777, 3720, and 3655 cm^–1^), even if the ν­(C≡N) peak was not observed with subsequent dosing. ν­(C≡N) of Al–OH species can be clearly seen for samples with low SiOHAl density (Figure S4), highlighting the preference of CD_3_CN to hydrogen bond with framework sites. In certain cases, such as for H^+^-MFI-12.0, more Al–OH peaks were added to improve the quality of fit that cannot be readily observed during successive dosing. For the rest of the samples, one partially hydrolyzed peak was used for an appropriate fit. Peak integration of Py IR features was performed through OPUS software (exact peak area, not from baseline) with limits set at 1565–1515 cm^–1^ for the Py-Brønsted acid site (BAS) peak and 1465–1535 cm^–1^ for the Py-Lewis acid site (LAS) peak (±3 cm^–1^).

Acid site densities (μmol g^–1^) were determined using the equation below based on calculated peak areas and integrated molar extinction coefficients (ε) for CD_3_CN and Py at 25 and 150 °C, respectively.^54,55^ ε for SiOHAl hydrogen bonding with CD_3_CN was adjusted from the zeolite FER to the MFI framework using the SiOHAl count of NH_4_^+^-MFI-12.0-NaEx at 25 °C, a defined sample containing 96% of framework sites as determined by the Na/Al ratio from ICP-OES. We also modified the molar extinction coefficient of partially hydrolyzed species that we divided by half as only one SiOHAl leads to one partially hydrolyzed site instead of two as is assumed by Wichterlová and coworkers. The assumption that two SiOHAl sites lead to one LAS is not well supported, and using ε for partially hydrolyzed sites leads to an underestimation of sites and the total acid/Al ratio. The rationale for adjusting ε for SiOHAl to the MFI framework is as follows. Thibault-Starzyk and colleagues used state-of-the-art thermogravimetry and FTIR spectroscopy to establish the most accurate ε for adsorbed pyridine in MFI (BAS = 1.09 and LAS = 1.71 cm μmol^–1^) where, in particular, they also show that the BAS ε depends on the zeolite framework.^57^ Pyridine molar extinction coefficient values from Emeis are an average of different zeolite frameworks and silica–aluminas, causing substantial differences in site counts (BAS = 1.67 and LAS = 2.22 cm μmol^–1^) that lead to lower acid/Al ratios and do not satisfy acid site mass balances.^58^ In this line, ε for SiOHAl was adjusted for the MFI framework as the work we referenced used zeolite ferrierite to determine ε for BAS. Final ε values used for all samples titrated with CD_3_CN are denoted in Figure S5.

1 $$\mathrm{Acid}\;\mathrm{site}\;\mathrm{density}\;(\mu \;mol\;g^{-1})=\left(\frac{IR\;peak\;area\;(cm^{-1})}{\varepsilon \left(cm\;\mu \;mol^{-1}\right)}\right)\times \left(\frac{Wafer\;area\;(cm^{2})}{g_{cat}}\right)$$

### 2.3 Reaction Measurements

#### 2.3.1 Propane Cracking Rate Constants

Propane cracking rate constants were measured without contributions of extrinsic dehydrogenation derived from carbonaceous deposits as described by Gounder and Iglesia.^59^ Zeolites in their NH_4_^+^ or H^+^ form (0.030–0.600 g) were sieved to 180–250 μm and loaded into a fixed-bed reactor equipped with a 12 mm OD × 8 mm ID quartz tube (Technical Glass Products) supported between quartz wool plugs (1 cm below and 1 cm above). The remaining reactor tube volume was filled with quartz chips (crushed and sieved to 180–425 μm particle size, Pyromatics) to minimize the contribution of gas phase reactions outside of the catalyst section. A small diameter immersion Omega K-type thermocouple probe (Inconel and sheathed in a 3 mm OD × 2 mm ID quartz tube thermocouple well) was placed in the center of the catalyst bed and used to control and monitor the furnace temperature. The reactor tube was loaded into a vertical split tube furnace (equipped with a 20 cm AmpCo bronze thermal block) of a Microactivity Effi Flow Reactor (PID Eng & Tech) with the catalyst bed positioned in the center. The furnace was located within a hot box maintained at 160 °C for all experiments. The gas feed composition and flow rates of propane (Airgas, research grade nonodorized C_3_H_8_), nitrogen (Airgas UHP N_2_), hydrogen (Airgas UHP H_2_), and oxygen (Airgas UHP O_2_) were controlled by individual mass flow controllers (Bronkhorst). The gas feed was preheated by flowing through a coiled tube located within the hot box prior to entering the reactor section in a top-down flow configuration. In all experiments, the pressure drop across the reactor length was below 0.1 bar. To remove carbonaceous deposits, samples were treated in a flowing stream of synthetic air (mixture of 21% Airgas UHP O_2_ and 79% N_2_; 50 sccm) and after a N_2_ purge (Airgas UHP N_2_; 50 sccm), in pure H_2_ (Airgas UHP H_2_; 50 sccm) at 530 °C for 1 h each (with a ramp rate of 10 °C/min). The catalyst was cooled to a reaction temperature (475 °C) in flowing N_2_ and held for 0.5 h. For reactions, a volumetric flow rate of 100 sccm with 5–15% propane and 20% H_2_ and balancing N_2_ were fed to the reactor. Propane conversions were kept between 0.5 and 2% during all screenings to minimize the extrinsic dehydrogenation function derived from carbonaceous deposits, where this was possible with adequate product pressures. A drain valve prevented excessive accumulation of liquids in the cold trap (although no liquids were observed). The reactor effluent outlet was analyzed via online gas chromatography (GC-2010*Plus*, Shimadzu) equipped with a Thermal Conductivity Detector (TCD) and a Flame Ionization Detector (FID) in parallel analytical lines. Hydrocarbon products were separated by a RTX-1 column (Restek) and quantified by FID. All reported rate constants and conversions (Figure S6) are an average of 5 GC measurements (133 min on-stream) where observed GC peak areas changed by less than 2%.

## 3 Results and Discussion

### 3.1 Textural Characterization and Observation of Water-Induced Changes in Al Coordination

#### 3.1.1 Physicochemical and Textural Characterization

Commercial MFI (ZSM-5), BEA, FAU, and CHA materials were obtained from Zeolyst, Tosoh, and ACS Material in either H^+^ or NH_4_^+^ form (Table 1). These catalysts are denoted throughout this work as *C*-*X*-*Y*-*T*, where *C* is the counterbalancing cation (H^+^ and NH_4_^+^), *X* is the framework code, *Y* is the Si/Al ratio measured with ICP-OES, and *T* is the temperature of NH_4_^+^-form activation or Na^+^ exchange in celsius (°C). The activation temperature was varied between 500 and 800 °C to produce steamed H^+^-MFI-12.0-T materials from NH_4_^+^-MFI-12.0 with varying site heterogeneity (i.e., different distributions of framework, partially hydrolyzed, and extraframework sites). For the sake of brevity, standard conditions (500 °C) and zeolite commercial vendors will be omitted throughout this work (information included in Section 2), as almost all catalysts are activated at standard conditions and each material has a different Si/Al ratio as measured with ICP-OES (Table 1). Furthermore, materials labeled as NH_4_^+^-form refer to *in situ* activated catalysts used directly prior to any reaction or analysis, while those labeled as the H^+^-form refer to *ex situ* activated catalysts that were stored under ambient laboratory conditions (25 °C, RH 20–50%). Examples of MFI material names for *in situ* and *ex situ* activated NH_4_^+^ form samples are NH_4_^+^-MFI-12.0 and H^+^-MFI-12.0–700 °C, respectively. In terms of a Na^+^ exchanged material, an example name is H^+^-MFI-12.0-NaEx 25 °C for a catalyst that has been ion-exchanged at room temperature (25 °C). To confirm the crystalline and microporous integrity of all zeolitic materials after standard and nonconventional oxidative thermal pretreatment, materials were verified with pXRD and Ar adsorption isotherms. PXRD patterns showed appropriate diffraction features for each zeolite framework (see Figure S7), while micropore volumes determined from Ar adsorption isotherms at −196 °C (see Table 1 and Figure S8) also provide support.

**Table 1:** Physicochemical Properties and Elemental Compositions of Commercial MFI, BEA, FAU, and CHA Zeolites

Vendor	Catalyst and measured Si/Al^a^	*V*_micro_ ^b^ (cm^3^ g^–1^)	*V*_meso_ ^c^ (cm^3^ g^–1^)	Si/Al^d^
Zeolyst	H^+^-MFI-12.0	0.159	0.036	11.5
	H^+^-MFI-12.0-600 °C	0.163	0.031	11.5
	H^+^-MFI-12.0-700 °C	0.157	0.050	11.5
	H^+^-MFI-12.0-800 °C	0.151	0.059	11.5
	H^+^-MFI-16.2	0.166	0.062	15
	H^+^-MFI-27.8	0.165	0.057	25
	H^+^-MFI-40.4	0.170	0.062	40
	H^+^-MFI-147	0.164	0.039	140
	H^+^-BEA-12.8	0.168	0.397	12.5
	H^+^-FAU-16.6	0.282	0.495	15
Tosoh	H^+^-MFI-11.4	0.173	0.066	11.5
	H^+^-MFI-12.2	0.161	0.069	11.5
ACS Material	H^+^-CHA-11.7	0.221	0.078	10

a Measured with ICP-OES.

b V_micro_ determined from the derivative of the semilogarithmic plot (∂(*V*_ads_)/∂(log­(*P*/*P*_o_)) vs log­(*P*/*P*_o_)) of an Ar adsorption isotherm (−186 °C).

c *V*_meso_ = *V*_total_ – *V*_micro_, where *V*_total_ is determined by the total volume adsorbed at *P*/*P*_o_ = 0.96.

d Provided by a commercial vendor.

From the literature, it is evident that framework Al sites can exhibit changes in coordination upon exposure to water that can lead to the formation of partially hydrolyzed and extraframework sites that influence rates and selectivity. Therefore, we focus on commercial NH_4_^+^-MFI-11.4 to avoid differences in catalyst crystallization and calcination protocols and gauge the inherent site heterogeneity. Figure 1 shows the OH region IR spectra of the hydroxyl groups present in NH_4_^+^-MFI-11.4 (bottom) and H^+^-MFI-11.4 (top), where we employ the former zeolite (NH_4_ activation *in situ*; 500 °C for 1 h) to observe undisturbed Al sites (i.e., the material was never exposed to water). Both samples exhibited an absorbance band between 3745 cm^–1^ (external Si–OH) and 3735 cm^–1^ (internal Si–OH), which is attributed to Si–OH groups on outer and inner zeolite surfaces. The 3610 cm^–1^ band for bridging hydroxyls (SiOHAl) is solely observed in NH_4_^+^-MFI-11.4, and additional bands of Al–OH groups (3777, 3720, and 3655 cm^–1^) are present in H^+^-MFI-11.4, where their nature and environment (i.e., partially hydrolyzed or extraframework) are highly debated in the literature.^5,32–34,37,60,61^

**Figure 1:**
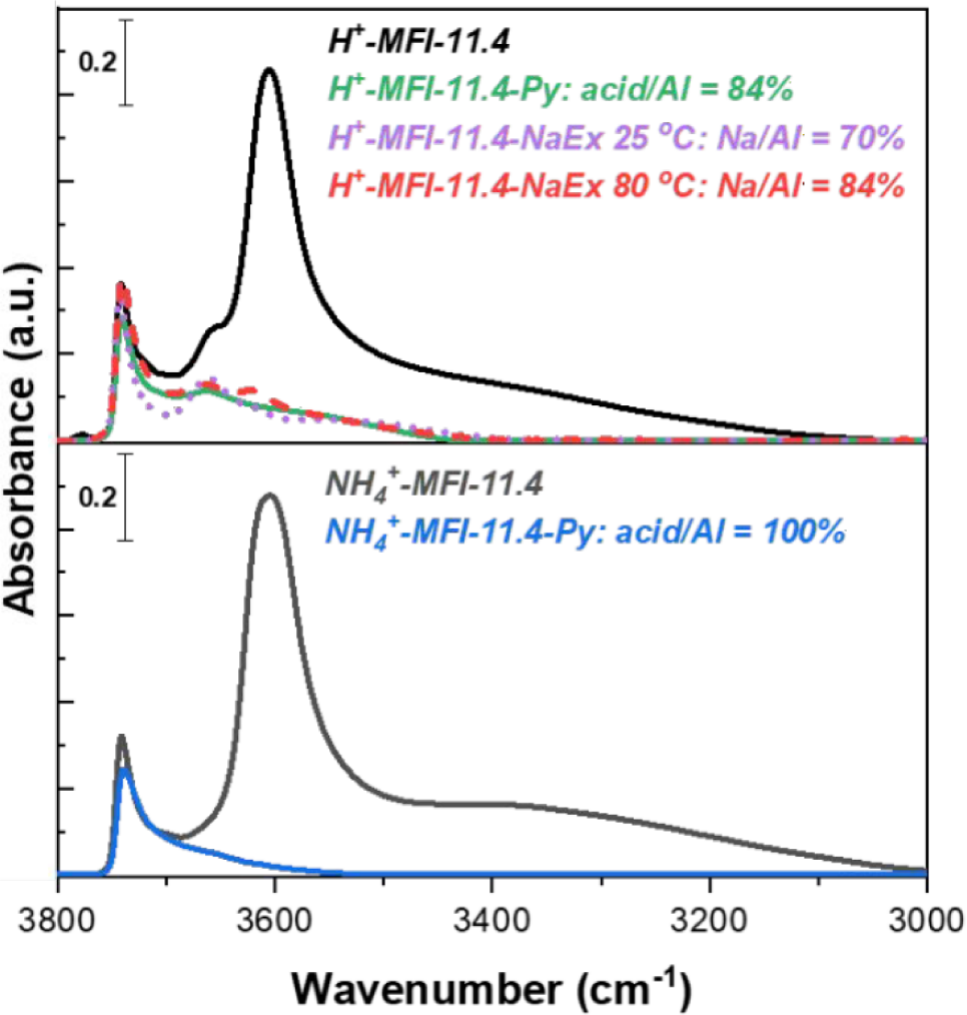
Transmission IR spectra of (bottom) NH_4_^+^-MFI-11.4 and (top) H^+^-MFI-11.4. IR spectra were collected at room temperature (25 °C) under vacuum (1 × 10^–4^ Torr) after in situ thermal dry air flow treatment at 500 °C for 1 h to obtain information on pristine OH stretches, after pyridine titration (C–H stretches removed for clarity) and Na ion exchange at 25 and 80 °C (1 M NaNO_3_, 24 h).

Materials were activated with the same air treatment protocol, indicating that Al–OH sites can be formed upon exposure to ambient water (25 °C and 20–50% RH), which has also been observed by van Bokhoven and coworkers for high-Al-containing FAU and MOR zeolites.^32,41,62,63^ The presence of Al–OH and Si–OH due to framework site hydrolysis for the *ex situ* NH_4_ activated zeolite led to a decrease in the broadband region between 3500–3000 cm^–1^, suggesting weaker hydrogen bonding interaction between proximal Al–OH/Si–OH and SiOHAl sites.^7^ Base titratability of Al–OH sites was gauged with pyridine (the most employed probe in the literature) and proper molar extinction coefficients^54^ to find that only 84% of Al sites are accounted for in H^+^-MFI-11.4, while all sites are accessible for NH_4_^+^-MFI-11.4 (Figure 1). Residual Al–OH bands were also observed after H^+^-MFI-11.4 was ion-exchanged at room temperature (1 M NaNO_3_, 24 h), where 70% of Al sites were Na^+^-exchanged as indicated by metal content analysis. Raising the ion exchange temperature to 80 °C led to an additional 14% increase of exchanged Al sites, yet Al–OH bands still persisted. In contrast, NH_4_^+^-MFI-11.4 was almost fully exchanged (96% of Al) after three Na ion exchanges at room temperature. The inability to ion exchange or titrate Al–OH sites with pyridine is typically attributed to the presence of nonacidic extraframework and partially hydrolyzed sites, yet, again, distinction between these sites is not established.^64^

We also provide support for the absence of nonacidic extraframework sites by titrating H^+^-MFI-12.0-NaEx 25 °C with ammonia, ethylamine, *n*-propylamine, iso-propylamine, and pyridine (Figure 2). For the last two bases, the remaining Al site speciation was fully titrated but with differing Brønsted and Lewis acid site counts. Furthermore, the total acid count decreased with decreasing base size and proton affinity, illustrating the weaker nature of these sites, in contrast to bridging hydroxyls. The ability to titrate 30% of nonexchanged sites suggests that the Al–OH group IR bands (3777, 3720, and 3655 cm^–1^) observed in H^+^-MFI-11.4 most likely correspond to partially hydrolyzed sites rather than true extraframework species. Consistent with this, White et al. used quantitative direct-excitation and sensitivity-enhanced ^27^Al ssNMR techniques at varying magnetic field strengths to show that dried H^+^-MFI zeolites (Si/Al = 12.0 and 16.2 from Zeolyst) do not exhibit detectable hexacoordinated extraframework Al species prior to significant hydrothermal exposure.^35,36^ In the following section, we aim to quantify the total acid count using different bases while referencing the Al content to examine the effect of base properties on site density measurements in systems containing either only SiOHAl sites or a mixture of SiOHAl and Al–OH sites.

**Figure 2:**
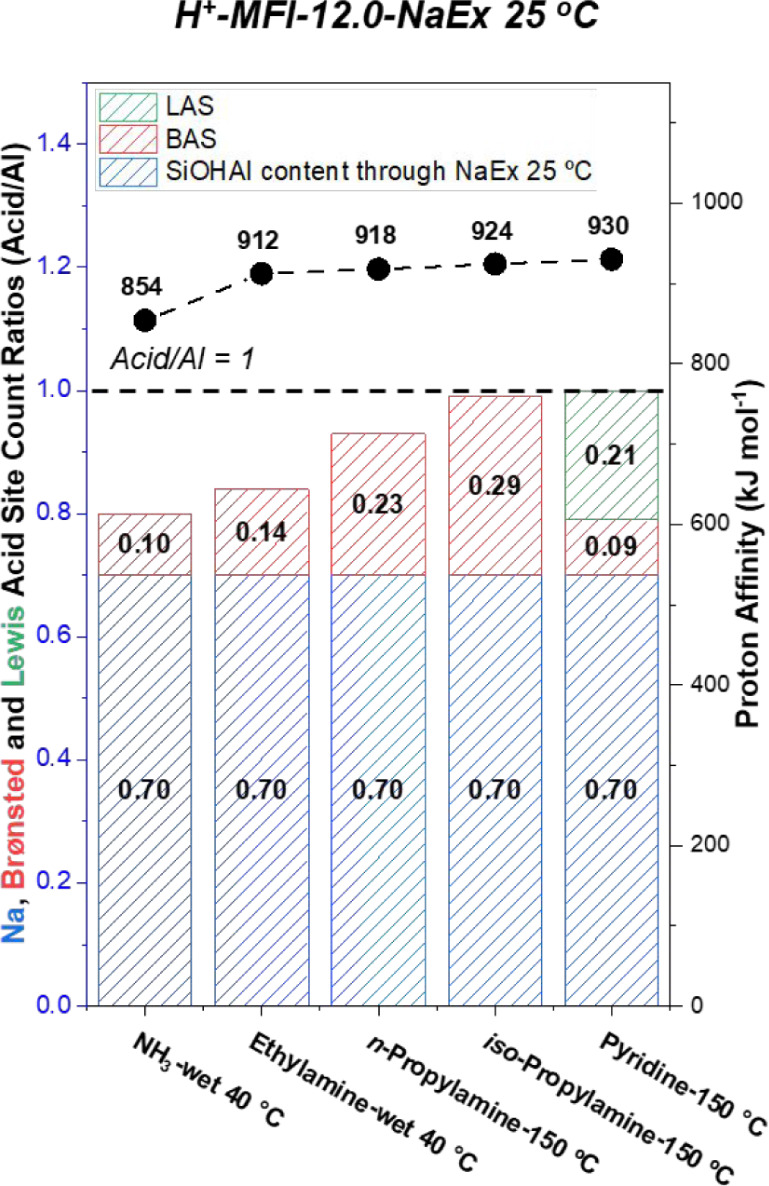
Na and Brønsted acid site (BAS) count ratios were determined with metal content analysis (ICP-OES) and transmission IR and temperature-programmed desorption (TPD) experiments for BAS and LAS determination of H^+^-MFI-12.0-NaEx 25 °C with bases of varying proton affinity (obtained from the NIST database) and size. Transmission IR spectra were collected at 150 °C under vacuum after in situ thermal dry air flow treatment at 500 °C for 1 h to obtain pyridine titration counts. TPD experiments were performed after Ar treatment at 500 °C for 1 h for NH_3_, ethylamine, *n*-propylamine, and iso-propylamine with their denoted dry or wet purge temperatures. Site count errors are within ≤5%.

### 3.2 Effect of Base Type, Proton Affinity, and Size on Brønsted and Lewis Acid Site Counts of NH_4_^+^ *-*MFI-11.4 and H^+^-MFI-11.4

Quantification of acid site counts is essential to describe and perform correlations with any acid–base reactions of interest catalyzed over solids.^6,7,65–66^ The type of base will affect the determined properties of the probed material; therefore, the literature precedent for selection is to choose a molecule that is more basic than acidic, can distinguish between Brønsted and Lewis acid sites, and has a size similar to the reactant to probe accessibility.^67–70^ Over the past 60 years, there has been a range of techniques available to quantify acid counts that in essence involve a base probe with infrared and ssNMR spectroscopy, as well as temperature-programmed desorption experiments.^57,71–75^ Nonetheless, we will omit protocols with ssNMR spectroscopy as they are not readily accessible to some catalysis laboratories. Even among the techniques being discussed and their advantages/limitations, the methods employed are chosen due to their availability. Infrared spectroscopy was the initial technique used to compare the OH bond frequencies of hydroxyl groups found in zeolites. Qualitative analysis of acid sites with IR became amenable with the use of probes such as pyridine, ammonia, acetonitrile, and CO. However, with the use of molar extinction coefficients for pyridine and acetonitrile, the former is predominantly employed for counting Brønsted and Lewis acid sites through identification of stretches associated with these sites.^54,76^

In the case of temperature-programmed desorption experiments, the method consists of saturating the surface of a dry catalyst with a suitable base probe and subsequently desorbing chemisorbed species with online mass spectrometer detection. The most used probe for TPD analysis is ammonia that desorbs intact, leading to debate on the interpretation of what types of sites are being counted in TPD experiments without the aid of spectroscopy. Most ammonia TPD profiles are broad peaks, especially for low Si/Al ratios, leading to convolution of peaks that are difficult to decouple.^77^ Selection of physisorbed base purge protocols can add confusion to TPD profiles as appropriate temperature and/or the assistance of water are required, both of which can influence acid counts.^42,43,78^ Furthermore, ammonia desorption peak positions in porous materials can be affected by base readsorption that can shift the position of peaks;^79,80^ therefore, a nuanced approach must be employed to make sure that these effects are mitigated as some research groups have done.^42,43^ Nonetheless, other probe molecules have been employed for TPD measurements such as alkylamines that undergo selective Hofmann elimination on Brønsted acid sites to form an alkene and NH_3_.^51,53,81^ Consequently, the Brønsted acid density can be determined through a 1:1 stoichiometry with the detected base or alkene.^43^ Yet, an underutilized quantity that can be determined from alkylamine TPD experiments is the Lewis acid count that corresponds to intact base desorption.

Therefore, here, we aim to quantify the total acid count with different bases while referencing Al content to see the effect of base properties on determined site densities in the sole presence of SiOHAl and a combination of SiOHAl and Al–OH sites (no extraframework species). We employ FTIR with CD_3_CN and pyridine and TPD methods with NH_3_ and alkylamines (Figures S3, S5, S9, and S11 contain representative TPD profiles and IR spectra) that involve dry and wet purge approaches that remove physisorbed species.^43,51,54,55^ Evaluating site counts of these bases for site quantification of NH_4_^+^-MFI-11.4 and H^+^-MFI-11.4 (Figure 3a,b) shows that the *in situ* activated NH_4_^+^-MFI-11.4 is insensitive to base proton affinity and size (for bases under consideration). This is in line with results obtained by Abdelrahman et al. for the same materials, which are rationalized by the primary presence of bridging hydroxyls (SiOHAl) that readily serve as proton donors.^51,82^ On the other hand, for H^+^-MFI-11.4, there is more variation of Brønsted and Lewis acid site counts and accessibility of total acid density due to the presence of Al–OH species. Pyridine can only titrate 84% of Al sites, while smaller bases such as deuterated acetonitrile and ethylamine can access all sites, potentially the same for the other alkylamines if the Lewis signal was calibrated. Differences in the proportion of Brønsted and Lewis counts seem to also depend, apart from the absence of ion-exchangeable sites (*i.e*., Na-titrated sample in Section 3.1), on base fit within the microporous structure as a lesser dependence is observed for the BEA framework (Figure S12). Nonetheless, NH_3_ titrations on average have a higher “Brønsted” site count than for other bases. The effect of base fit and higher “Brønsted” ammonia counts can be further exemplified with nonmicroporous γ-Al_2_O_3_ that solely contains Al–OH species (Figure 3c). For this material, the trend of increasing Brønsted counts and proton affinity can be observed without the influence of base size, illustrating the weaker nature of these sites as was observed in H^+^-MFI-12.0-NaEx 25 °C and in the literature for phosphorus-modified zeosils.^82^ Furthermore, NH_3_ adsorbs on Al–OH sites that are not fully displaced by water (even at higher temperatures; Figure S13), accentuating the unselective titration of ammonia and raising questions about what this base titrates on zeolites. Moreover, what is noteworthy about γ-Al_2_O_3_ is that pyridine only counts Lewis acid sites on this material even though it has hydroxyl groups and it is employed for hydrocarbon reforming reactions in the petrochemical industry where its acid role is debated.^83,84^ Overall, it is clear that distinguishing between Brønsted and Lewis sites in a solid acid system and assigning this role in reactions of interest is challenging when only using denotations given by base titrations.

**Figure 3:**
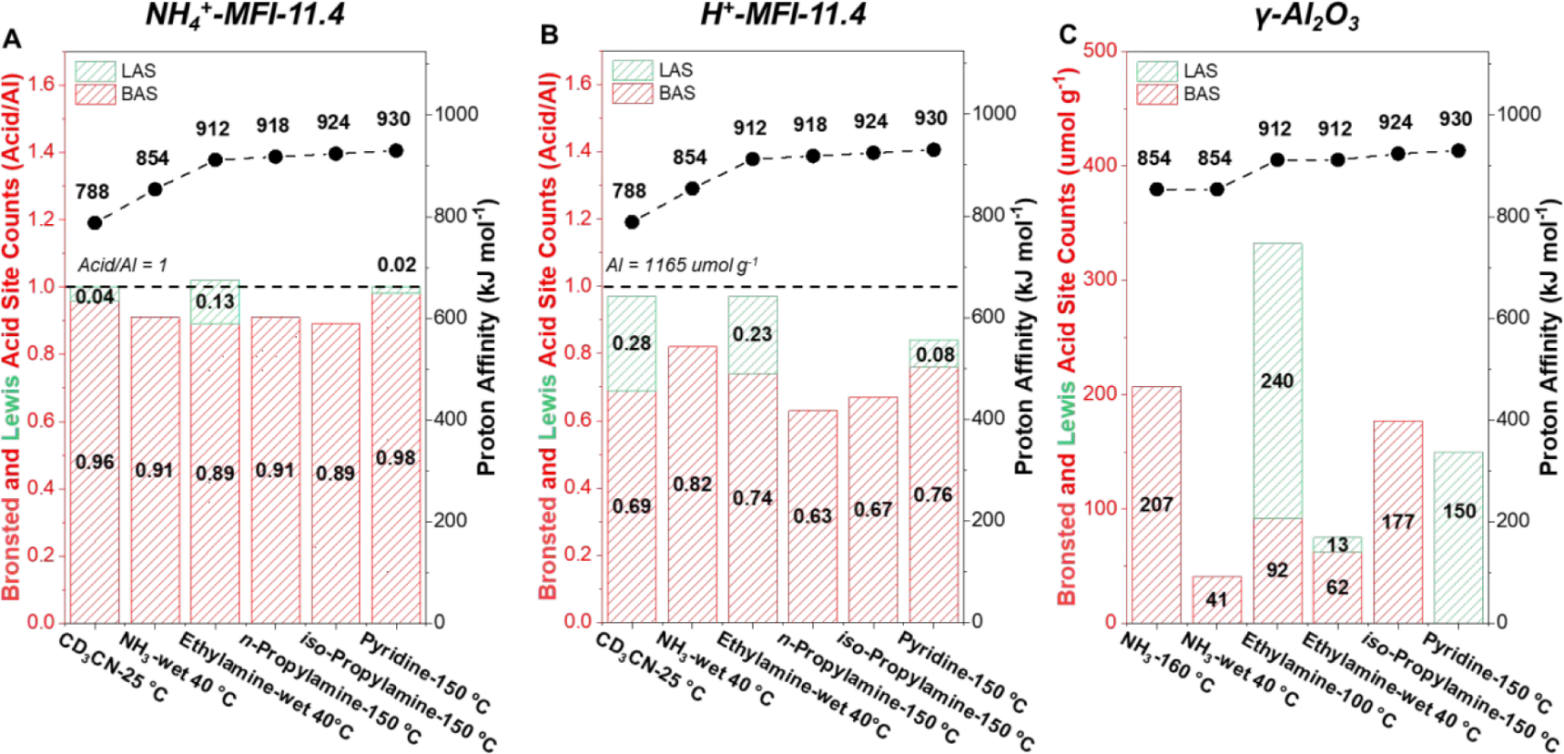
Brønsted and Lewis acid site ratios or counts determined with transmission IR and temperature-programmed desorption (TPD) experiments of (A) NH_4_^+^-MFI-11.4, (B) H^+^-MFI-11.4, and (C) γ-Al_2_O_3_ with bases of varying proton affinity (obtained from the NIST database) and size. Transmission IR spectra were collected after in situ thermal dry air flow treatment at 500 °C for 1 h of NH_4_^+^-MFI-11.4, H^+^-MFI-11.4, and γ-Al_2_O_3_ to determine pyridine (150 °C) and CD_3_CN (25 °C) titration counts under a vacuum. TPD experiments were performed after Ar treatment at 500 °C for 1 h for NH_3_, ethylamine, *n*-propylamine, and iso-propylamine with their denoted dry or wet purge temperatures. Site count errors are within ≤5%.

### 3.3 Determination of Lewis Acid Site Counts: The Challenge of Displacing Intraporous Base-Extended Hydrogen-Bonded Networks

Even though Brønsted and Lewis acid site counts vary with the selected base, not all probes are made equal. Zeolites that only contain SiOHAl sites (SiOHAl/Al → 1) show Brønsted acid site count insensitivity to base proton affinity and size (for bases under consideration) serving as an important reference point. Therefore, the opposite, deviations of Brønsted and Lewis acid site counts that vary with the selected base, provide a quantitative indication that site speciation has changed in addition to the observation of Al–OH bands in IR spectra. Ethylamine and deuterated acetonitrile are at least of appropriate size and could access all of the sites in NH_4_^+^-MFI-11.4 and H^+^-MFI-11.4, including Al–OH species; therefore, we focus on their applicability for materials with varying Si/Al ratios and site heterogeneity. In this section, we concentrate on TPD methods for ethylamine, the smallest alkylamine that can undergo Hofmann elimination and access the porous structure of an 8-membered ring zeolite such as CHA.^85^ We leave the discussion of deuterated acetonitrile IR for the following section, where we will examine it in detail and with reference to counting partially hydrolyzed sites in Sn-β.

When generalizing site titrations to other Si/Al ratios of commercial MFI Zeolyst samples (nominal Si/Al of 140, 40, 25, and 15), ethylamine has its pitfalls with TPD measurements. Dry and wet purge experiments vary in effectiveness in their removal of physisorbed base molecules when proton density is decreased, leading to under- or over-quantification of Lewis acid sites (Figure 4) as observed for Sn-β.^56^ Over-quantification can be understood by behavior observed for alcohols of different chain lengths by the Martens group that was ascribed to additional adsorption on siloxane bridges and silanol nests at low proton densities that form extended hydrogen-bonded networks that are difficult to displace with or without water.^86^ This is demonstrated by the required physisorbed base purge temperature that is dependent on the amount of Brønsted acid sites, as for Sn-β lower purge temperatures are required for ammonia and alkylamines.^56^ Non-Brønsted-containing catalysts can have higher Si/Metal ratios before overcounting is observed; nonetheless, it is important to note that the purge temperature is also influenced by the amount of Si–OH defects. Increasing the dry or wet purge temperature circumvents over-quantification; nonetheless, sites are now undercounted, as shown Figure 4a,b. This suggests that ethylamine interacts with Al–OH and Si–OH species with similar strengths, causing unwanted desorption of the former with dry purge approaches and through potential changes in coordination when employing a wet purge.

**Figure 4:**
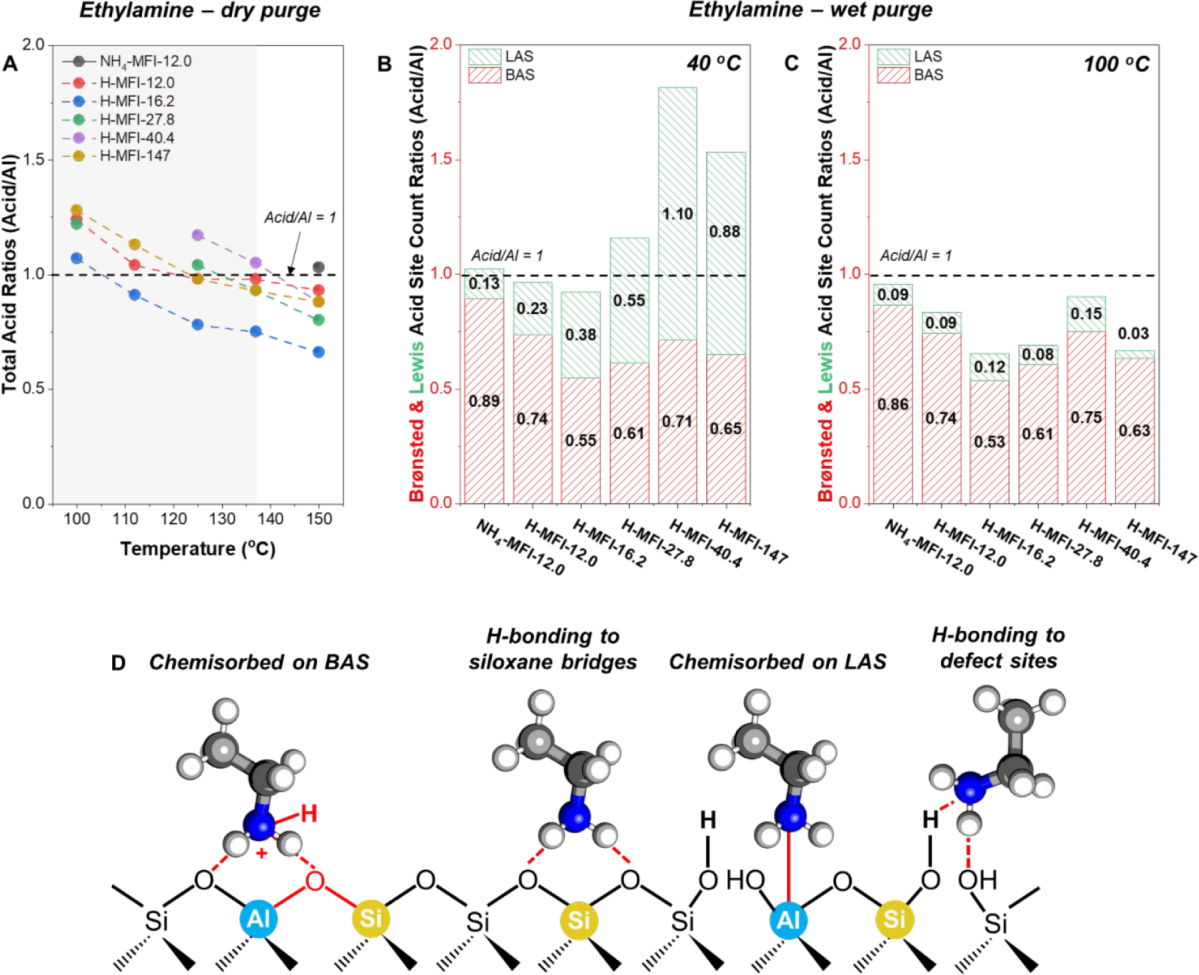
Total acid and Brønsted, and Lewis acid site count ratios determined through ethylamine temperature-programmed desorption (TPD) experiments with (A) dry purge between 100 and 150 °C and wet purge at (B) 40 and (C) 100 °C of commercial MFI zeolites of varying Si/Al ratios. TPD experiments were performed after Ar treatment at 500 °C for 1 h with denoted dry or wet purge temperature. (D) Illustration of different modes of amine adsorption on zeolites that includes chemisorption on Brønsted and Lewis acid sites and hydrogen bonding to siloxane bridges and H-bonding species such as silanols and Al–OH groups (adapted from ref 87). It is important to note that the bridging hydroxyl of partially hydrolyzed groups was omitted for clarity. Site count errors are within ≤5%.

The motivation for applying a wet purge approach originated from researchers at ExxonMobil.^52^ In the late 1990s, they demonstrated that Lewis-bound ammonia could be removed by showing agreement between the NH_3_ TPD profile and the hydrated ^27^Al ssNMR spectrum of NH_4_^+^-MFI, which was produced from the H^+^-form using two methods: ion exchange and gas-phase saturation with a wet purge at 125 °C. The agreement in acid site counts (even though subtotal Al) and the absence of hexacoordinated Al in NH_4_^+^-MFI when compared to H^+^-MFI led them to conclude that only Brønsted acid sites give rise to the highest temperature desorption peaks (325–430 °C) in the TPD experiments. Gounder and coworkers provided an extension of this concept as they were able to desorb ammonia from Cu Lewis acid sites in exchanged zeolites that satisfied a site balance with respect to the parent zeolite that contained a H^+^/Al ratio of 0.65.^42^ Measured residual H^+^ sites in Cu-exchanged CHA samples (Si/Al = 4.5, Cu/Al = 0–0.20) in combination with metal content determination confirmed that two H^+^ sites were exchanged by a Cu^2+^ ion, as expected, to maintain framework charge neutrality. Nonetheless, recent work by White et al. provides clarity on tetrahedral site speciation in aluminosilicates, commonly assigned to H^+^, through quantitative direct-excitation and sensitivity-enhanced ^27^Al NMR techniques. They posited that depending on the extent of catalyst hydration, the apparent amount of extraframework sites change intensity, while the signal for partially hydrolyzed Al­(IV) sites separates or converges with the signal of framework Al­(IV).^35,36^ In the case of hydrated samples, tetracoordinated partially hydrolyzed and framework Al site signals overlap. Therefore, we suggest that the perceived agreement between ion exchange and gas phase NH_3_ wet purge experiments (although acid/Al < 1) is most likely because both count ion-exchangeable sites that include some hydrolyzed species. This interpretation can be rationalized by the ability of NH_3_ to adsorb on the Al–OH sites in γ-Al_2_O_3_ (Figure 3). From our measurements, we see parity between NH_3_ wet purge, Na^+^, and NH_4_^+^ ion exchange at 80 °C for unsteamed commercial MFI zeolites (Figure S14). Nonetheless, this observation is probably framework-dependent, as the temperature of ion exchange depends on zeolite structure (Figure S15). Only when all nonframework Al extraction is performed through (NH_4_)_2_SiF_6_ treatment are SiOHAl sites solely observed in 2D ^27^Al multiple-quantum magic-angle spinning ssNMR spectra of H^+^-MFI-16.2 with a 40% decrease in partially hydrolyzed sites that matches well with our Brønsted acid site counts for this material.^33,35,36^ Lercher and colleagues performed (NH_4_)_2_SiF_6_ treatment on Si/Al > 25 samples, which led to a decrease in rate, resulting in a linear pentane cracking rate vs SiOHAl correlation that resembled unsteamed samples synthesized by Exxon.^5,28^ From all the observations mentioned, it is apparent that the water-induced dynamic nature of partially hydrolyzed species and the weak nature of some of these species make it difficult to quantify and account for the total aluminum content.

### 3.4 Deuterated Acetonitrile as a Probe to Quantify Site Heterogeneity—Framework, Partially Hydrolyzed, and Extraframework Sites

A subset of partially hydrolyzed sites appear to be sensitive to the presence of water, making them unable to undergo ion exchange and titration with wet purge TPD methods. Furthermore, the interaction of bases with Al–OH and Si–OH species is somewhat similar in strength such that even with dry purge approaches that depend on proton density, unwanted desorption occurs, leading to acid/Al < 1. The base titrant of choice for partially hydrolyzed sites of tetravalent metals such as Sn is CD_3_CN, and through a Lewis adduct, it can distinguish between partially hydrolyzed (2320–2312 cm^–1^) and fully incorporated closed sites (2308 cm^–1^) through shifts in the ν­(C≡N) stretching frequency, as established in the literature, while accounting for gas phase acetonitrile (2265 cm^–1^).^56,87^ Consequently, CD_3_CN titration experiments do not require extensive purge steps (typically at 25 °C under vacuum) to remove the physisorbed species. When applied to aluminosilicates, CD_3_CN exhibits bands at 2300–2297 and 2330–2310 cm^–1^ that are ascribed to SiOHAl and partially hydrolyzed acid sites, respectively.^55,88–90^ Moreover, in contrast to other employed bases that undergo protonation, acetonitrile hydrogen bonds to SiOHAl sites in a selective manner.^88^ The ability to differentiate OH species through hydrogen bonding also allows to distinguish between Al–OH (2285 cm^–1^) and nonacidic Si–OH groups (2275 cm^–1^).^89^

Therefore, a complete description of the active site distribution in aluminosilicates with no extraframework species, as shown in Figure 2a,b, can be obtained with CD_3_CN from a perspective that does not involve base protonation and extensive purge protocols. Here, we take advantage of the ability of CD_3_CN to parse out SiOHAl, partially hydrolyzed, and extraframework sites obtained from a total site balance to monitor the effect of NH_4_^+^-form activation (*ex situ*) on the formation of these sites in NH_4_^+^-MFI-12.0. However, several considerations for peak fitting are delineated to minimize the uncertainty of peak deconvolution due to simultaneous titration of SiOHAl (2300–2297 cm^–1^) and partially hydrolyzed (2330–2310 cm^–1^) acid sites and peak overlap. To identify sites present in NH_4_^+^-MFI-12.0 and H^+^-MFI-12.0, infrared spectra were collected with increasing CD_3_CN coverage at 25 °C (Figure 5) until gas-phase CD_3_CN was observed, indicating saturation of all adsorption sites. At low CD_3_CN coverage, the peak center of the first dose (2320–2312 cm^–1^ at <0.3 CD_3_CN/Sn) is used as a criterion to determine the presence of open sites in stannosilicates.^91^ For our aluminosilicate samples, in contrast, the first dose had peak centers between 2330 and 2310 and 2300–2297 cm^–1^ for SiOHAl and partially hydrolyzed sites, respectively, where successive doses did not lead to shifts in peak centers until the presence of gas phase acetonitrile (2265 cm^–1^). The lack of a peak center shift with dosing highlights that the first dose criterion is not as essential for the set of MFI samples employed; however, performing progressive dosing is still useful to confirm the presence of sites before significant overlap occurs. After complete sample saturation (observance of 2265 cm^–1^), the IR spectrum was deconvoluted into its principal component peaks centered at 2330–2310 and 2300–2297 and fixed at 2285, 2275, and 2265 cm^–1^ (additional details in Section 2) to determine the density of sites using eq 1 with integrated molar extinction coefficients obtained by Wichterlová et al.^55^ Nonetheless, the molar extinction coefficient of SiOHAl was adjusted with NH_4_^+^-MFI-12.0 that is a defined sample with 96% of framework sites (Figure S5). We also modified the molar extinction coefficient of partially hydrolyzed species that we divided by half as only one SiOHAl leads to one partially hydrolyzed site instead of two as was assumed in their work (Section 2 contains the rationale for adjusting molar extinction coefficients).^55^ Peak deconvolution of H^+^-MFI-12.0 (Figure S3) without including Al–OH species (2285 cm^–1^) led to an overestimation of SiOHAl and acid/Al > 1, in contrast to counts determined by other bases. A better estimate of SiOHAl counts was determined by including the Al–OH species (2285 cm^–1^) on samples that exhibit Al–OH bands (3777, 3720, and 3655 cm^–1^) even though its ν­(C≡N) peak is not observed with successive dosing. ν­(C≡N) of Al–OH species can be clearly observed for samples with low SiOHAl density (Figure S4), exemplifying the preference of CD_3_CN to hydrogen bond to framework sites. In certain cases, such as for H^+^-MFI-12.0, other Al–OH peaks are added to improve the quality of fit that cannot be readily observed during successive dosing but were observed in aluminosilicates.^55^

**Figure 5:**
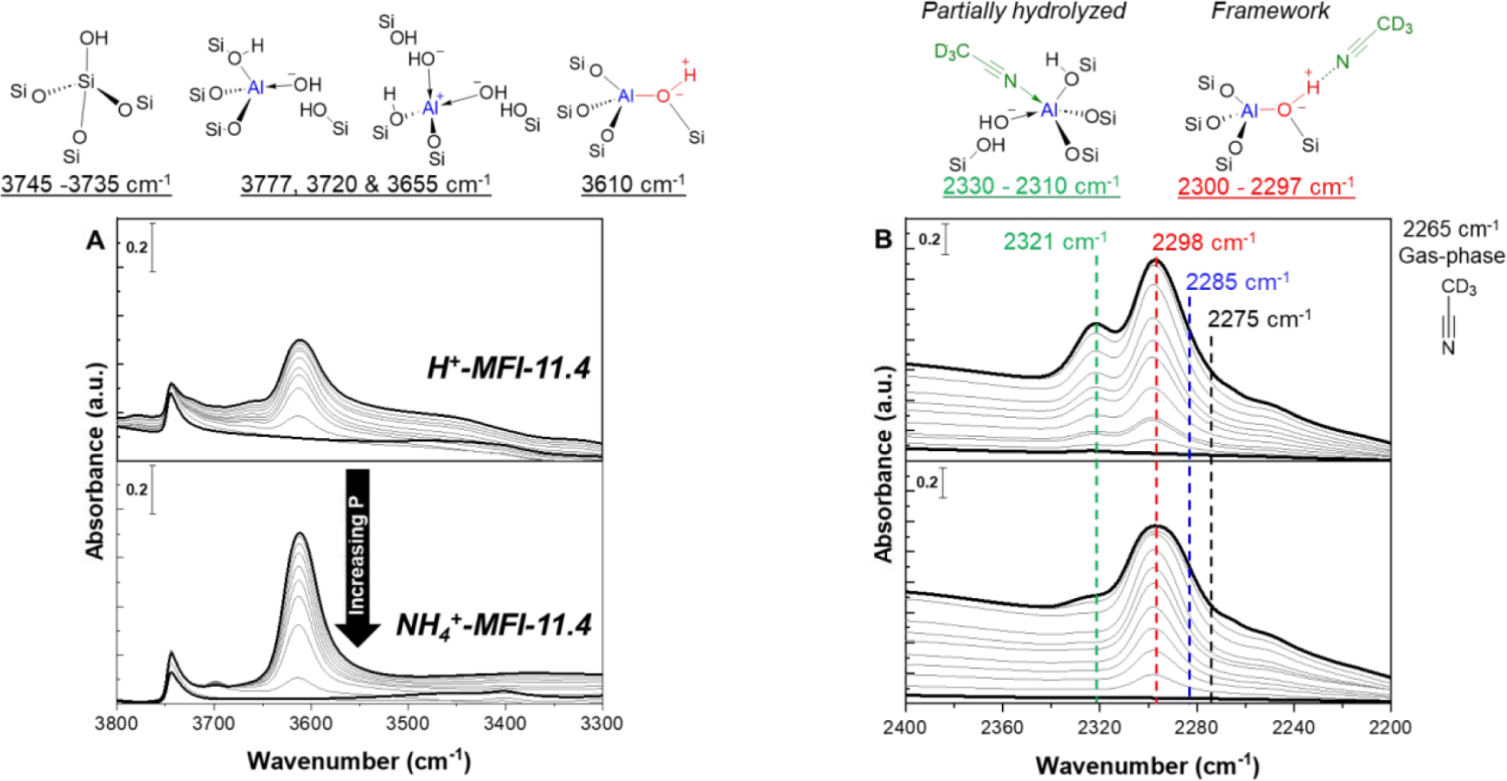
Transmission IR spectra of (A) OH and (B) C≡N regions for (bottom) NH_4_^+^-MFI-11.4 and (top) H^+^-MFI-11.4 materials with increasing CD_3_CN coverage at room temperature (25 °C). (A) OH stretches and structures are shown for silanol groups (3745–3735 cm^–1^), partially hydrolyzed sites (3777, 3720, and 3655 cm^–1^), and framework SiOHAl (3610 cm^–1^). (B) C≡N stretches and structures of adsorbed CD_3_CN on partially hydrolyzed sites (2330–2310 cm^–1^), framework SiOHAl (2300–2297 cm^–1^), Al–OH groups (2285 cm^–1^), silanol groups (2275 cm^–1^), and gas-phase CD_3_CN (2265 cm^–1^) are shown with dashed lines. Saturated CD_3_CN difference IR spectra (bold line). Transmission IR spectra were collected after in situ thermal dry air flow treatment at 500 °C for 1 h of NH_4_^+^-MFI-11.4 and H^+^-MFI-11.4 to determine CD_3_CN (25 °C) titration counts under vacuum. Site count errors are within ≤5%.

As previously discussed, the exact structures of partially hydrolyzed Al sites remain under debate.^32,33^ However, experimental and theoretical ^27^Al ssNMR studies suggest that these sites may resemble [(SiO)_4__–__*n*_-M­(OH)_*n*_], as found in Sn-β.^34–36^ Still, there is no consensus on whether the bridging hydroxyl remains.^37^ In addition, the location of hydroxyl groups within the zeolite and their extent of hydrogen bonding influence their IR stretching frequencies.^38^ As such, accurate modeling of the structure of all partially hydrolyzed sites and the corresponding ν­(C≡N) shifts of adsorbed CD_3_CN, which appear in the 2330–2310 cm^–1^ range, remains a challenging task. Regarding extraframework sites, they are generally believed to exist as oxides, hydroxides, or multinuclear clusters, though their precise structures are still not well understood.^39,40^ An important boundary condition for distinguishing partially hydrolyzed species from extraframework ones is the reversible transition between octahedral and tetrahedral coordination upon hydration and dehydration.^35,41^ Comparable strategies have been applied to Sn-β, where the characterization of dehydrated materials, followed by hydration treatments, helps identify framework-incorporated Sn species. In contrast, extraframework SnO_*x*_ and SnO_2_ species are unaffected by such dehydration–rehydration cycles.^92,93^ White and coworkers applied this concept using quantitative ^27^Al ssNMR on dried H^+^ -MFI-12.0 and H^+^ -MFI-16.2 (both from Zeolyst) to demonstrate that hexacoordinated extraframework Al species do not exist prior to significant hydrothermal exposure.^35,36^ For these samples, hexacoordinated sites reappeared only upon hydration. As shown here, the full aluminum site distribution, including partially hydrolyzed sites, can be captured using CD_3_CN titration of both NH_4_^+^-MFI-12.0 and H^+^-MFI-12.0. Only when materials are steamed under harsher ammonium activation protocols do we observe a decrease in the Lewis acid site density (Figures 6C and S16) and Al–OH stretches (Figure 6D). We suggest this change results from the formation of extraframework species, even though their exact structure is still under debate.

**Figure 6:**
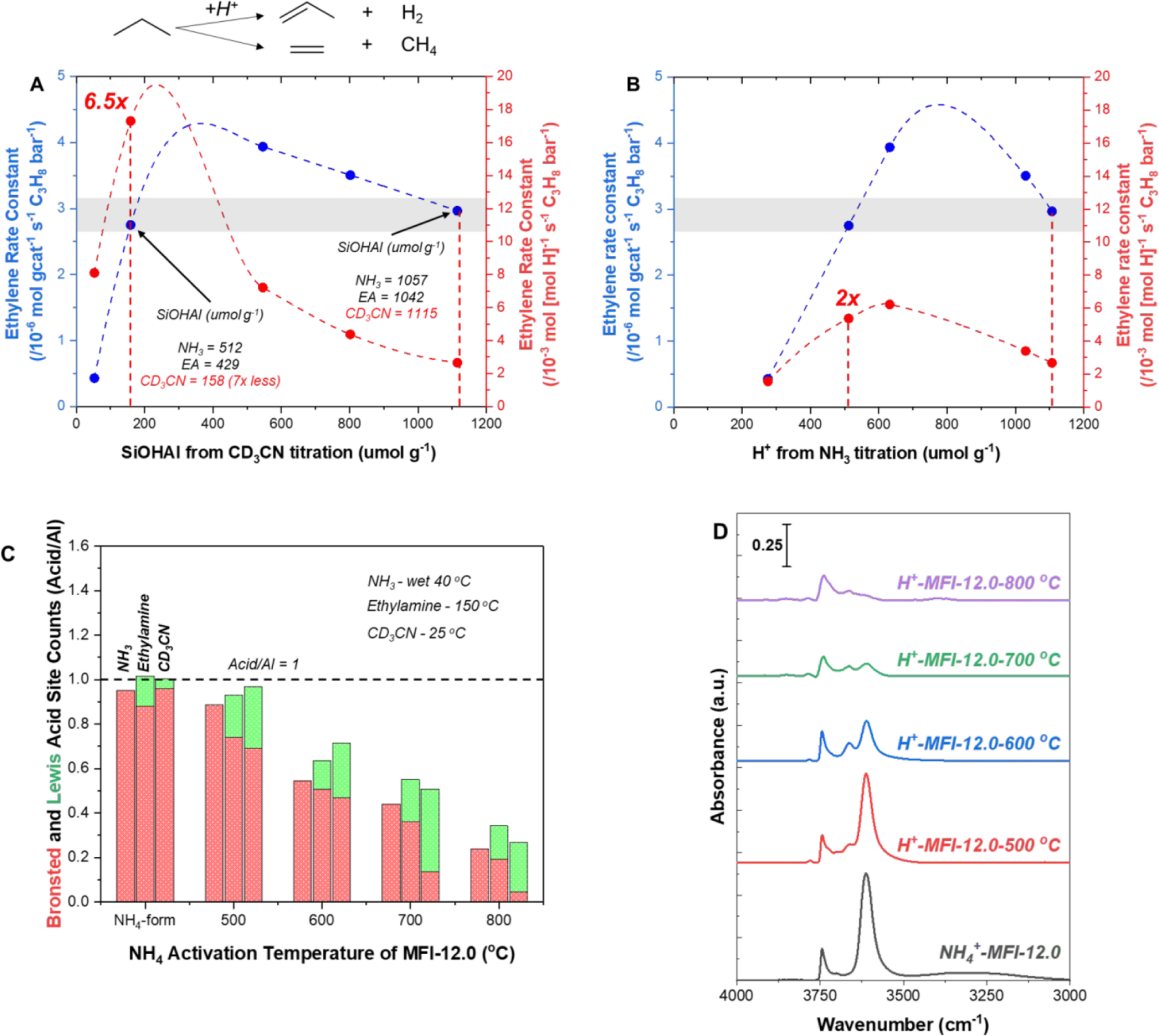
Propane cracking rate constant per g_cat_ and per (A) SiOHAl and (B) H^+^ as obtained by CD_3_CN and ammonia titration, respectively. (C) Brønsted and Lewis acid site ratios determined with transmission IR and temperature-programmed desorption (TPD) experiments measured for NH_4_^+^-MFI-12.0 treated with different ex situ NH_4_ activation temperatures. (D) OH region transmission IR for NH_4_^+^-MFI-12.0 treated with different ex situ NH_4_ activation temperatures. Propane cracking rate constants were determined at 475 °C as described by Gounder and Iglesia et al., in ref 59. Propane (Airgas, research grade C_3_H_8_) and H_2_ (Airgas UHP H_2_) were diluted in N_2_ (Airgas UHP N_2_) to concentrations of 5 to 15% and 20%, respectively, at a volumetric flow rate of 100 sccm (differential conversions). Transmission IR spectra were collected at 150 and 25 °C under vacuum after in situ thermal dry air flow treatment at 500 °C for 1 h to obtain pyridine CD_3_CN titration counts, respectively. TPD experiments were performed after Ar treatment at 500 °C for 1 h for NH_3_ and ethylamine with 40 °C wet and 150 °C dry purge temperatures (no SiOH influence), respectively. OH region transmission IR spectra were collected at 25 °C under vacuum after in situ thermal dry air flow treatment at 500 °C for 1 h. Site count errors are within ≤5%.

### 3.5 Determination of Bridging Hydroxyl-Catalyzed Propane Cracking Rate Constants

After we evaluated “Brønsted” and “Lewis” acid site titration methods, we aim to further contextualize ammonia, ethylamine, and CD_3_CN site counts for extraframework quantification with normalizing propane cracking rate constants over steamed NH_4_^+^-MFI-12.0. Materials were prepared by varying the *ex situ* NH_4_^+^ activation temperature between 500 and 800 °C with Drierite dried air, where a combination of adsorbed water from storage and inherent Al site stability facilitated site hydrolysis. This treatment protocol was adapted from Yashima et al. and used to produce steamed H^+^-MFI-12.0 materials with varying site heterogeneity and no detectable structural degradation (as described in Section 3.1).^44^ In their work, they were able to cause hydrolysis of SiOHAl sites that improved the dealumination efficiency of zeolite Mordenite while preserving its crystalline and microporous integrity. Propane cracking reactions (5 to 12.7 kPa C_3_H_8_ with a 20 kPa cofeed of H_2_, 475 °C) were performed with H^+^-MFI-12.0 materials of varying site heterogeneity as described by Gounder and Iglesia to minimize the influence of extrinsic dehydrogenation functions derived from carbonaceous deposits.^59^ Forward rate constants of ethylene were calculated as described in their work in the protolytic range (C_2_H_4_:CH_4_ carbon ratio 2:1) using a 20 kPa cofeed of H_2_ and considering formalisms of approach-to-equilibrium that were minimal for the conditions employed. Zeolites in their NH_4_^+^- and H^+^-forms were treated under air and pure hydrogen pretreatments that, in combination with 20 kPa cofed H_2_, mitigated initial CH_4_ and propylene insets caused by extrinsic dehydrogenation functions. Reactions were performed where the rates of formation of ethylene were measured as a function of time on stream and remained steady to determine cracking rate constants (Figure S5). In particular, protolytic events were conserved when conversions were kept below 2%, but with enough conversion to maintain adequate product alkene pressures that also mitigate extrinsic contributions. Measured rate constants were normalized by “Brønsted” acid site counts that are typically ascribed to bridging hydroxyls, given that these sites are posited in the literature to catalyze rate-determining C–C and C–H bond-breaking steps of short alkanes (≤C_6_).^94,95^ Therefore, rate constants normalized to “Brønsted” acid sites should serve as a kinetic descriptor that reflect the effect of structural changes of framework sites on partially hydrolyzed and extraframework species. The presence of these in combination with framework bridging hydroxyls was found to enhance alkane cracking rates through entropic transition state stabilization.^5–9^

In this section, the goal is to contextualize acid site titrations and the observations reported by Exxon and Lercher to differentiate between framework, partially hydrolyzed, and extraframework sites of steamed materials. In Figure 6c, total acid site counts for reference NH_4_^+^-MFI-12.0 (NH_4_^+^ activation *in situ*; 500 °C for 1 h) are similar for all bases with slight differences in Brønsted and Lewis acid counts for ethylamine and CD_3_CN. As *ex situ* NH_4_ activation temperature increases, total acid counts decrease with CD_3_CN accounting for a greater amount, while Brønsted site counts remain comparable up to the 600 °C sample. After this temperature, CD_3_CN titrates more Lewis acid sites at similar total acid site densities between all bases, suggesting that base protonation through interaction with Brønsted sites is affected by the surrounding species for steamed materials, as observed for hydrocarbon cracking. Based on NH_3_ acid counts, H^+^-MFI-12.0-700 °C and H^+^-MFI-12.0-800 °C should still have half and a fourth of the protons of NH_4_^+^-MFI-12.0, respectively, while the SiOHAl IR signal (Figure 6d) for the mentioned steamed materials has low intensities, suggesting overcounting, leading to lower cracking rates per H^+^. A similar unselective titration argument can be used for ethylamine. Therefore, we propose that CD_3_CN is able to more accurately capture the identity of the site distribution of SiOHAl, partially hydrolyzed, and extraframework (by subtraction of total Al) sites without the obfuscation of base protonation. When rate correlations are taken into consideration with SiOHAl acid counts determined through CD_3_CN (Figure 6a) and propane cracking rate constants (measured as described by Gounder et al.^59^), the enhancement in rate can be captured and ascribed to the formation of “Lewis acidic” partially hydrolyzed and nonacidic extraframework sites, while the decrease in performance is due to the loss of SiOHAl. In contrast, the ammonia site counts (Figure 6b) fail to rationalize the cracking rate enhancement and decrease observed due to unselective titration of sites in steamed materials. With the zeolites considered in this evaluation, the sole effect of partially hydrolyzed sites on propane cracking rates cannot be definitively decoupled as extraframework sites can partially occlude microporous voids influencing rates through steric confinement.^96,97^ Moreover, in terms of Brønsted acidic protons of partially hydrolyzed sites, they were found by Crossley and colleagues to catalyze hexane cracking at a lower rate (in contrast to SiOHAl and SiOHAl–AlOH) by employing MFI zeolites with different extents of Na exchange.^9^ However, which hydroxyl of the partially hydrolyzed site is catalyzing the rate is an open question in the literature. As mentioned previously, modeling partially hydrolyzed sites will require knowing the positioning of hydroxyl groups in zeolites, as the bond angles can influence their IR OH stretches and hence their ability to catalyze hydrocarbon cracking.

From the results obtained on base selection for propane rate normalization and the proposed nature of rate enhancement through transition state stabilization by nonframework sites, it is evident that the acid function of solids cannot be described by a single parameter. Attempts to correlate hydrocarbon reactions with site counts of bases inherently assume that the stability of the carbocation will correlate with the energetics of formation for the ion-pair complexes formed by strong bases. Gorte and other groups are major proponents of this concept, as solids such as zeolites are materials with various hydroxyl groups in discrete environments that impart location-dependent solvation (e.g., van der Waals and hydrogen bonding with lattice oxygen of the porous structure) interactions with adsorbed molecules.^66,72,98–100^ Molecular-level acid–base descriptions of reaction turnovers become even more complex when zeolites are exposed to industrial conditions involving water (e.g., during regeneration). This leads to the creation of nonframework sites, which, when located near framework bridging hydroxyls, are believed to help stabilize transition states.^5–9^ In contrast, acids in aqueous solutions form a single species that, through Brownian motion, experiences an average solute–solvent environment, resulting in homogeneous solvation effects that can be described by a single parameter such as p*K*_a_. Therefore, one possible solution for establishing rate correlations with solid catalysts is to use a probe molecule that interacts with the catalyst in a manner similar to that of the actual reactant. For example, alkylamines can be used to titrate acid sites and correlate with reactions such as alkylamine Hofmann elimination or alcohol dehydration, an approach that has been reported in the literature.^51,101^ The rationale behind this strategy is that the adsorption complex formed between an alkylamine and a Brønsted acid site generates the same or a similar ion pair (B + ZOH → HB + ZO), where Coulombic attraction between the ions dominates the energetics. This interaction occurs similarly in both titration experiments and catalytic reactions conducted under comparable environments.^70^ Using a base probe similar to the reactant serves well for “Lewis acid” quantification in solids as limited approaches are available for characterization of these sites. Solid acids can possess a distribution of hydroxylated sites that can have varying degrees of Brønsted and Lewis behavior such that for the latter, the adduct is more difficult to describe due to the multiple types of Lewis interactions that can take place. When base titrations are extended to hydrocarbon reactions, there is a mismatch between the nature of adsorbed complexes. Hydrocarbons are weaker bases, although olefins are more basic than alkanes; as a result, they form a looser ion pair where bonding interactions with the zeolitic structure become more essential.^102^ The adsorption of an alkane leads to a pentacoordinated carbonium ion, which is fairly unstable and kinetically relevant at low conversions, requiring stabilization from van der Waal stabilization with lattice oxygen of the porous structure.^18,94^ In this regard, we propose that it would be better to use a probe reaction and focus on quantifying the identity of the sites (instead of ascribing Lewis and Brønsted character with a base) and their distribution throughout the zeolite structure to perform rate correlations with a set of catalysts.

### 3.6 Summary and Outlook on Quantifying Site Heterogeneity in Microporous Aluminosilicates

Framework SiOHAl sites can undergo coordination changes when exposed to water, leading to the formation of partially hydrolyzed and extraframework sites, which can significantly impact reaction rates and selectivity. Unlike acids in aqueous solutions, zeolites are solid materials containing hydroxyl groups in distinct porous environments. These environments create solvation interactions that vary by location, such as van der Waals forces and hydrogen bonding with lattice oxygen, which are specific to adsorbed molecules. As a result, it is crucial to choose the appropriate titration method to avoid unwanted structural changes and prevent probe-specific interactions that could obscure the relationship between structure and performance. This is demonstrated with propane cracking rate correlations performed in Section 3.5, where we suggest focusing on quantifying the identity and distribution of sites throughout the zeolite framework and correlating structure to a probe reaction, rather than attributing Lewis and Brønsted acid function to sites with a base. Nonetheless, it is important that the site distribution of the catalyst is consistent during site titration and reaction evaluations. For example, in NH_4_^+^-MFI-11.4 activated *in situ* under standard conditions, exposure to water after activation results in partially hydrolyzed sites. Therefore, if gas-phase evaluations are performed with NH_4_^+^-MFI-11.4, the catalyst’s site count should be determined using the NH_4_^+^-form with the same treatment procedure. The same catalyst activation rationale applies to zeolites that are used in their proton form. However, for proton form zeolites, storage conditions can become a factor in modifying site speciation, although this is dependent on the zeolite framework and aluminum content.^41,103^ Here, using a desiccator, controlling lab conditions, and periodically determining acid site distribution would increase confidence in site determination. Nonetheless, if a zeolite is used in water-containing reactions, analysis of the spent material may be necessary.

In terms of base titration method selection, for MFI materials considered in this study with Si/Al ≥ 15, Brønsted acid site counts do not vary with the selected base or ion exchange (Figure 7a); however, variation was observed for high aluminum content (Si/Al of 11.5), steamed materials, or specific framework types (as shown in Figure 7b). Furthermore, zeolites containing only SiOHAl sites (SiOHAl/Al → 1) exhibit Brønsted acid site counts that are insensitive to the base’s proton affinity and size, serving as a key reference point. Thus, the opposite, variations in Brønsted and Lewis acid site counts with the selected base, indicate that site speciation has changed, which can also be confirmed by observing Al–OH bands in IR spectra. Among all the base probes tested, ethylamine and deuterated acetonitrile are suitably sized and able to access all sites in NH_4_^+^-MFI-11.4 and H^+^-MFI-11.4, making them amenable for total acid site evaluations. However, some partially hydrolyzed sites are sensitive to the presence of water, preventing them from undergoing ion exchange and titration using wet–dry purge TPD methods. As a result, complete characterization of the active site distribution in aluminosilicates can only be achieved using CD_3_CN, as adsorption does not involve base protonation, and extensive purge protocols are not required. Therefore, our work provides a detailed titration strategy that allows for the quantitative determination of site heterogeneity in aluminosilicates, accounting for different site distributions (framework, partially hydrolyzed, and extraframework sites) and Al content without catalyst modification, while considering physisorbed species, base type, and size. We emphasize that assessing the whole site distribution (i.e., site heterogeneity) is important, as it can help explain why Pt supported on steamed and acid-treated USY exhibits Pt gradients within zeolite crystals^104^ and can aid in characterizing the inherent micro- and mesoscopic gradients formed during zeolite synthesis.^105^

**Figure 7:**
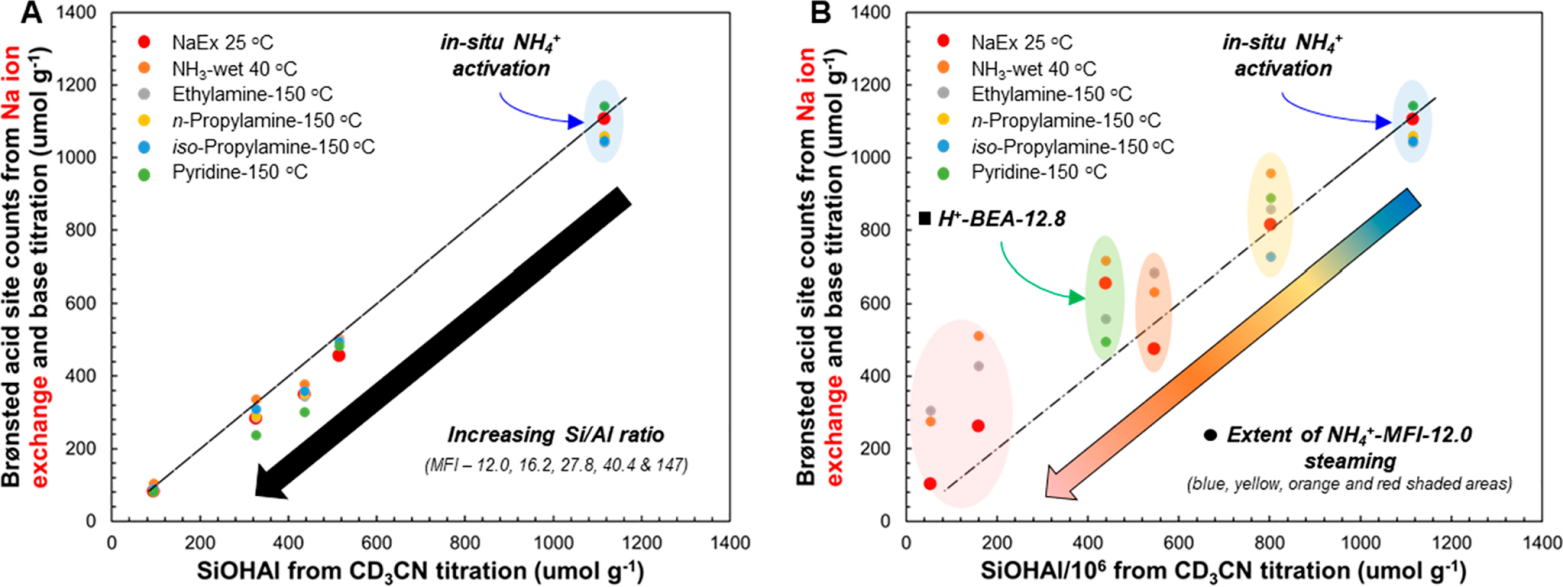
(A and B) Parity plot of Brønsted acid site (BAS) counts determined by Na ion exchange at 25 °C and acid site titrations versus SiOHAl counts determined by CD_3_CN for (A) commercial H^+^-MFIs of varying Si/Al and (B) NH_4_^+^-MFI-12.0 treated with different ex situ NH_4_ activation temperatures (NH_4_^+^-MFI-12.0 and H^+^-BEA-12.8 included as references of framework differences and the effect of in situ NH_4_^+^ activation at 500 °C, blue and green shaded areas, respectively). Na and BAS counts were determined with metal content analysis (ICP-OES) and transmission IR and temperature-programmed desorption (TPD) experiments. Transmission IR spectra were collected at 150 and 25 °C under vacuum after in situ thermal dry air flow treatment at 500 °C for 1 h to obtain pyridine CD_3_CN titration counts, respectively. TPD experiments were performed after Ar treatment at 500 °C for 1 h for NH_3_, ethylamine, *n*-propylamine, and iso-propylamine with their denoted dry or wet purge temperatures. Site count errors are within ≤5%.

## 4 Conclusions

Framework bridging hydroxyls are water-sensitive sites that can undergo hydrolysis, leading to the formation of nonframework species. These sites influence short alkane cracking rates in the kinetic regime, as demonstrated in this work. This effect is illustrated using commercial MFI aluminosilicates (ZSM-5) with varying site heterogeneity and Si/Al ratios. Their site distributions were quantified through a combination of temperature-programmed desorption and FTIR protocols, and the results were correlated with the propane cracking rates. IR characterization of commercial MFI-11.4, often used for zeolite studies across the literature, after NH_4_^+^-form activation exhibits the presence of Al–OH bands, particularly not only with exposure to wet air and increasing temperature but even with exposure to ambient conditions. A quantitative assessment of site heterogeneity was performed on *in situ* activated NH_4_^+^-MFI-11.4 and *ex situ* activated H^+^-MFI-11.4, showing that the former, solely containing framework sites, is insensitive to base proton affinity and size. On the other hand, H^+^-MFI-11.4 with Al–OH species has more variation in Brønsted and Lewis acid site counts. However, ethylamine and deuterated acetonitrile can quantify the whole acid site distribution for both catalysts. When generalizing site titrations to other Si/Al ratios of commercial Zeolyst samples (nominal Si/Al of 140, 40, 25 and 15), ethylamine has its pitfalls with TPD measurements, as wet purge experiments are required to remove physisorbed base that become ineffective at 40 °C when proton density is decreased, leading to over quantification of Lewis acid sites (that is quantified with ethylamine desorption). Similar behavior was observed in the literature for alcohols of different chain lengths that was ascribed to additional adsorption on siloxane bridges and silanol nests that form extended hydrogen bonded networks that are difficult to displace with water. Increasing the wet purge temperature to 100 °C circumvents overquantification, but sites are now undercounted as shown for H^+^-MFI-11.4 (NH_4_ activation *ex situ*; 500 °C for 1 h) compared to a purge temperature of 40 °C. When compared to site quantification with CD_3_CN, the lack of extensive purge protocols and the CN group can capture the distribution of sites in a zeolite as partially hydrolyzed sites form a Lewis adduct and framework sites hydrogen bond to acetonitrile instead of through Brønsted acid–base interaction that involves protonation and can be influenced by van der Waals interactions and proximity. Furthermore, underquatification with wet purges and variation in BAS and LAS counts is exemplified with gamma-Al_2_O_3_. When rate correlations are performed with CD_3_CN acid counts and propane cracking rate constants, the enhancement and decrease in rate can be ascribed to the formation of nonframework sites and loss of SiOHAl, respectively. Nonetheless, overall, Brønsted acid site counts do not vary with the selected base for MFI materials considered in this study with Si/Al ≥ 15; however, variation was observed for high aluminum content (Si/Al of 11.5), steamed materials, or specific framework types. In summary, the present work provides a nuanced titration strategy on how to quantitatively determine the site heterogeneity of aluminosilicates with differing site distribution (i.e., framework, partially hydrolyzed, and extraframework sites) and Al content without catalyst modification and with considerations of physisorbed species, base type, and size. We also reinforce literature observations of how water can induce changes in Al coordination during hydrothermal treatments and even at ambient conditions, especially in high Al containing materials, before catalysis, which leads to deviations in titration counts and adds variability in rate measurements. These observations and strategies should be extendable to other acidic zeolites and present ways to determine the site heterogeneities of materials in their dried state in an accessible manner that can serve as a starting point to evaluate structure–performance relationships.

## Supplementary Material



## Notes

Additional details of the IR experimental setup including schematics, supporting site quantification (IR and TPD), and reaction and textural characterization (XRD and physisorption) (PDF)
