# Supplementary material for: Quantifying Site Heterogeneity in Microporous Aluminosilicates and Implications for Catalysis
Source: ACS Catal. 2025 Oct 3;15(20):17314–32. doi: 10.1021/acscatal.5c01948 (PMC12538554; doi:10.1021/acscatal.5c01948)
Supplement: Supplementary file 1 [file cs5c01948_si_001.pdf]

Supporting Information

Quantifying Site Heterogeneity in Microporous Aluminosilicates and Implications for Catalysis

Edgard A. Lebrón-Rodríguez,[1] Fillipp E. Salvador,[2] Zahra Alikhani,[2] Jerome M. Evans,[2] Levi Callahan,[2] Nicole K. Mitchell,[1] Chenyao Huang,[2] Sudipta Ganguly,[2] Faysal Ibrahim,[2] Ive Hermans[1,2,3]*

[1]*Department of Chemical and Biological Engineering, University of Wisconsin-Madison, Madison, WI 53706, United States*

[2]*Department of Chemistry, University of Wisconsin-Madison, Madison, WI 53706, United States*

[3]*The Wisconsin Energy Institute, University of Wisconsin−Madison, Madison, WI 53726, United States*

*hermans@chem.wisc.edu

S.1. Transmission IR dosing setup and cell schematic

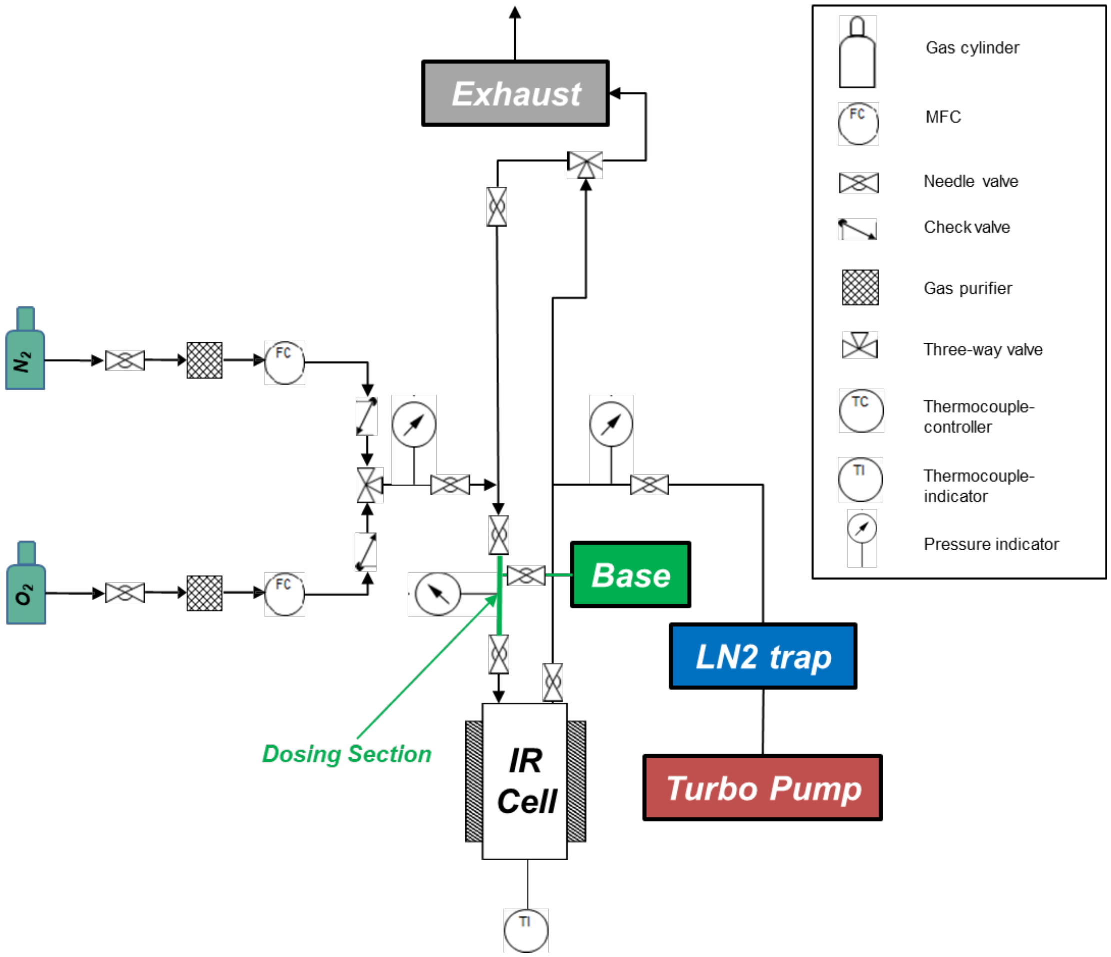

Figure S1: *Schematic illustration of IR dosing setup.*

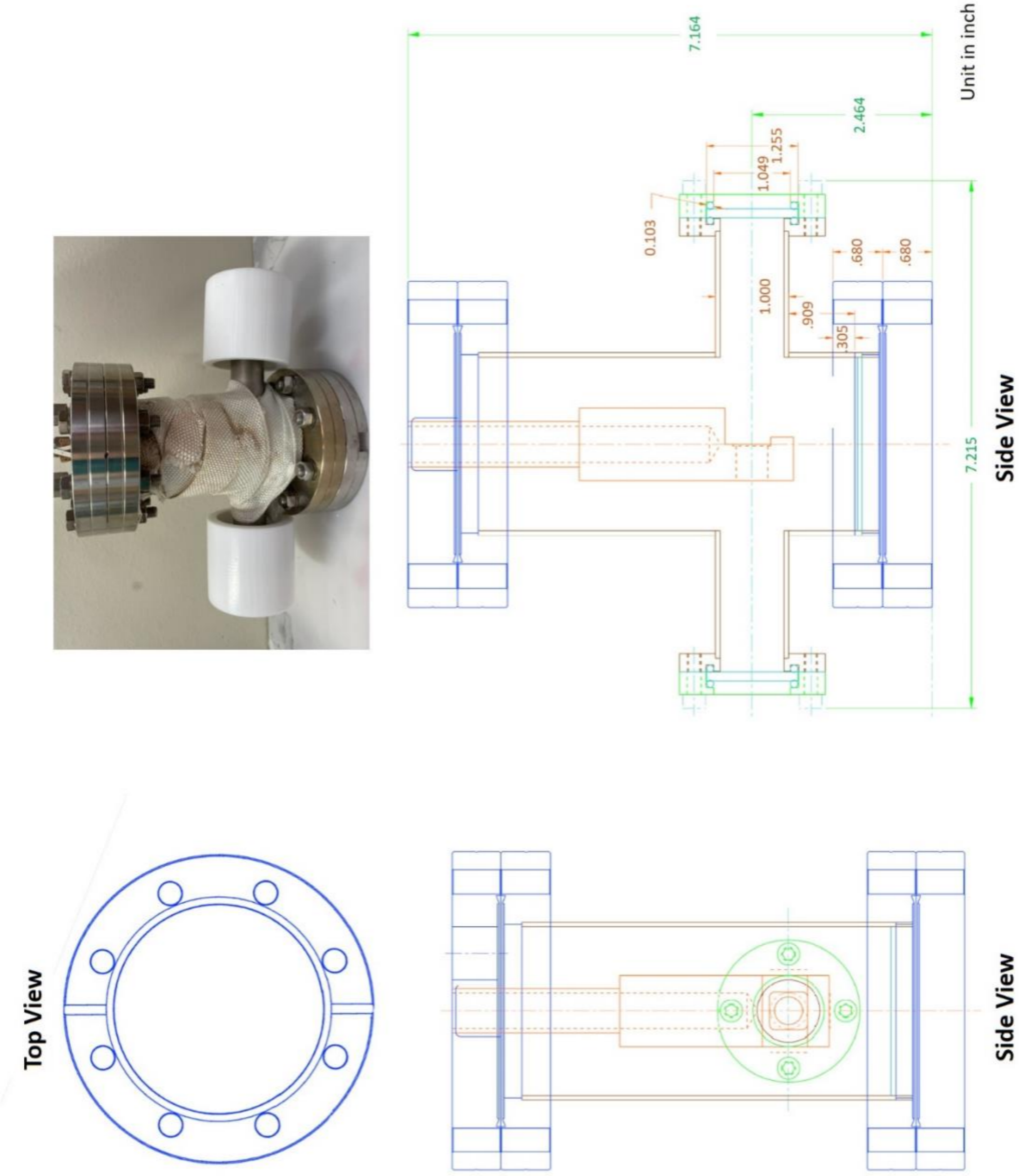

Figure S2*: Schematic illustration of custom IR cell.*

S.2. Supporting CD_3CN transmission IR spectra

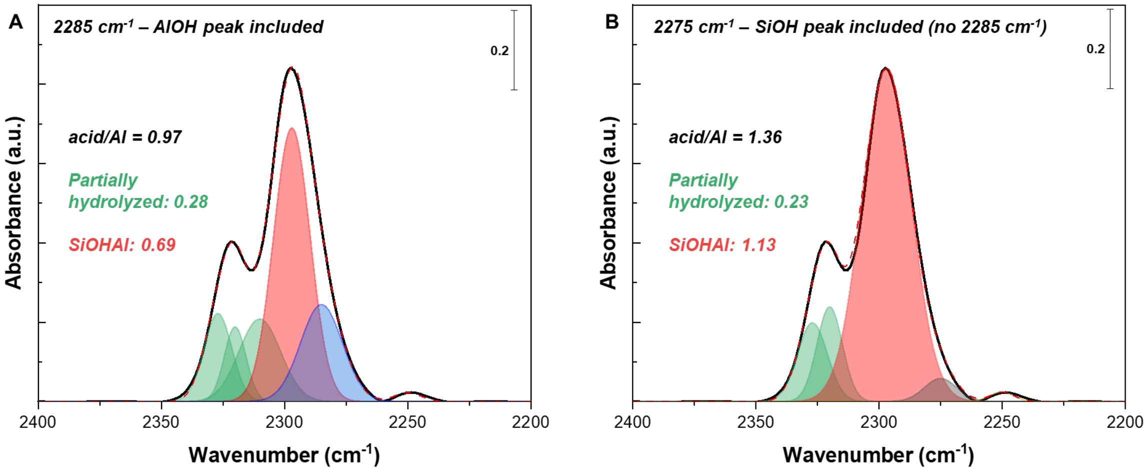

Figure S3: *Saturated CD_3CN transmission IR difference spectra of H^+-MFI-12.0 at room temperature (25 °C) comparing Al site distribution through deconvolution with (a) 2285 cm^{-1} Al-OH group peak included, and (b) the absence of 2285 cm^{-1} Al-OH group peak while including 2275 cm^{-1} Si-OH groups. Transmission IR spectra were collected after in-situ thermal dry air flow treatment at 500 °C to determine CD_3CN (25 °C) titration counts under vacuum. In general, saturated CD_3CN difference IR spectra were deconvoluted into partially hydrolyzed sites (2330 - 2310 cm^{-1}), framework SiOHAl (2300 - 2297 cm^{-1}) and Al-OH groups (2285 cm^{-1}) after 1h desorption.*

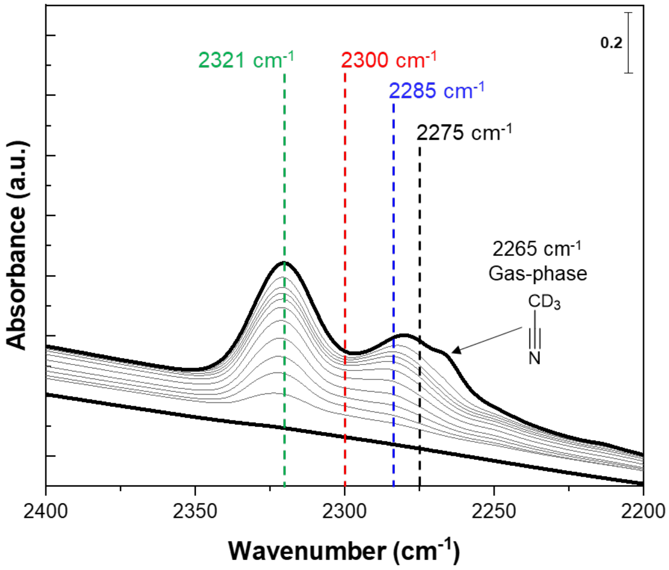

Figure S4: *Transmission IR difference spectra of H^+-MFI-12.0-800 °C at room temperature (25 °C) with increasing CD_3CN coverage where dashed reference lines are shown for partially hydrolyzed sites (2330 - 2310 cm^{-1}), framework SiOHAl (2300 - 2297 cm^{-1}), Al-OH groups (2285 cm^{-1}), silanol groups (2275 cm^{-1}), and gas-phase CD_3CN (2265 cm^{-1}). Transmission IR spectra were collected after in-situ thermal dry air flow treatment at 500 °C to determine CD_3CN (25 °C) titration counts under vacuum.*

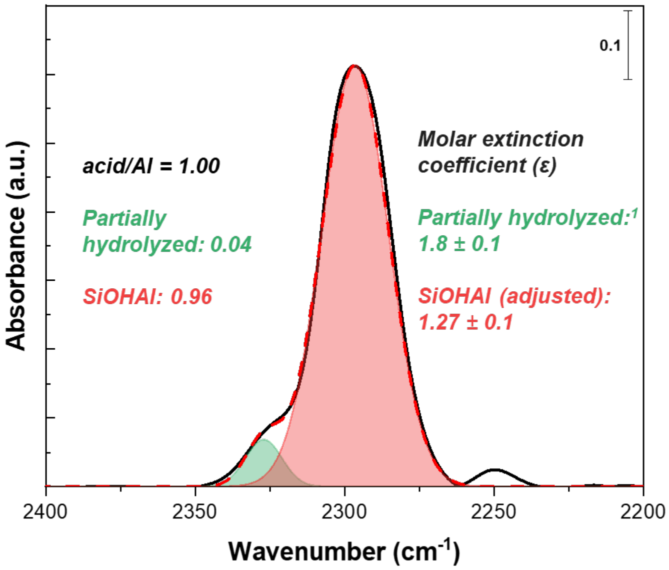

***Figure S5**: Saturated CD_3CN transmission IR difference spectra of NH_4^+-MFI-12.0 at room temperature (25 °C). Transmission IR spectra were collected after in-situ thermal dry air flow treatment at 500 °C to determine CD_3CN (25 °C) titration counts under vacuum and readjust the ε for SiOHAl hydrogen bonding with CD_3CN using NH_4^+-MFI-12.0, a defined sample containing 96% of framework sites. We also modified the molar extinction coefficient of partially hydrolyzed species that we divided by half as only one SiOHAl leads to one partially hydrolyzed site instead of two as it was assumed in Wichterlová et al. Here, saturated CD_3CN difference IR spectra was deconvoluted into partially hydrolyzed sites (2330 - 2310 cm^{-1}) and framework SiOHAl (2300 - 2297 cm^{-1}) after 1h desorption.*

S.3. Supporting reaction measurements of Propane cracking rates

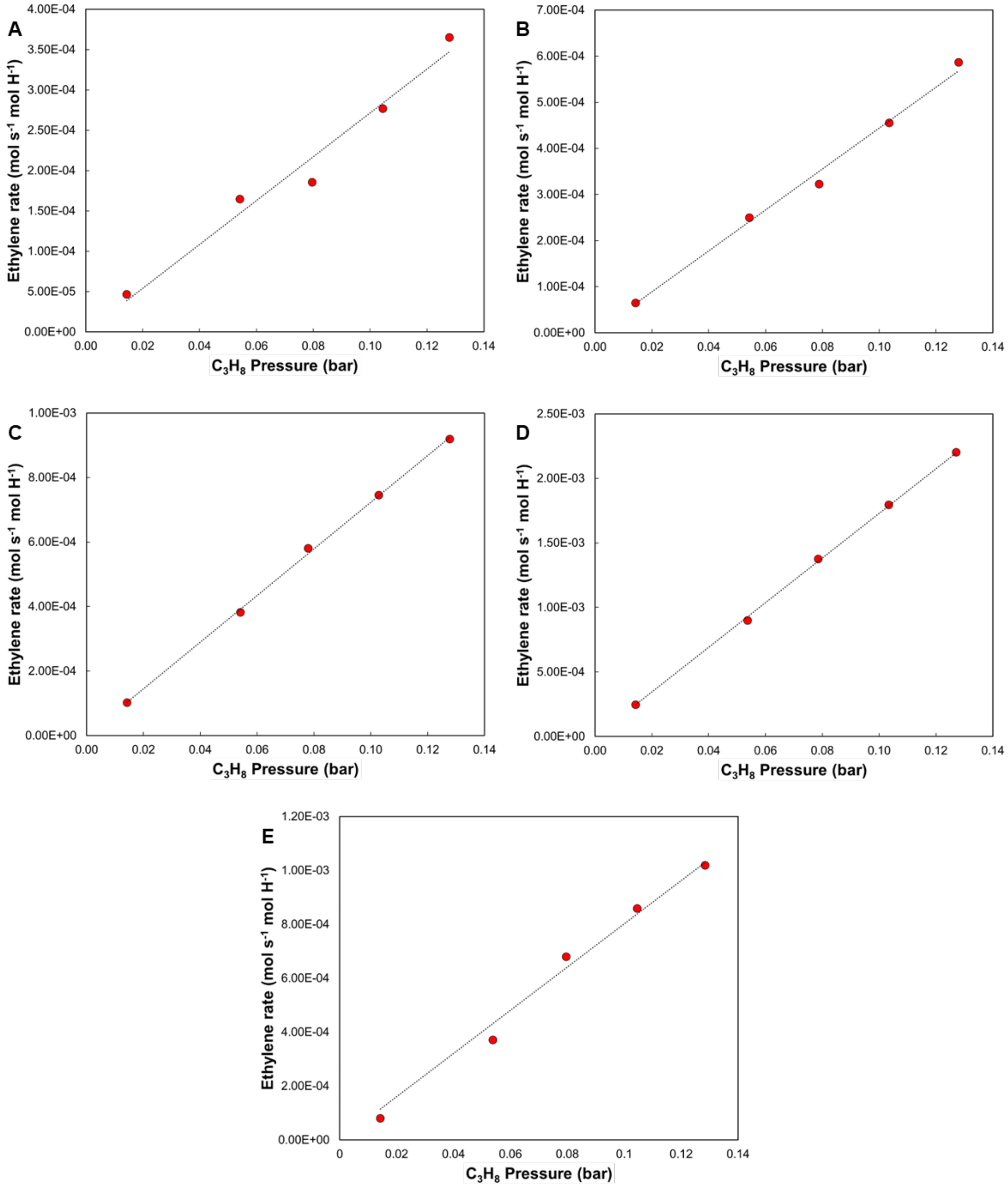

***Figure S6**: Propane cracking rate per SiOHAl obtained by CD_3CN as function of propane pressure (bar) for (a) NH_4^+-MFI-12.0, (b) H^+-MFI-12.0, (c) H^+-MFI-12.0-600 °C, (d) H^+-MFI-12.0-700 °C and (e) H^+-MFI-12.0-800 °C. Propane (Airgas, research grade C_3H_8) and H_2 (Airgas UHP H_2) were diluted in N_2 (Airgas UHP N_2) to concentrations of 5 to 15% and 20%, respectively, at a volumetric flow rate of 100 sccm (differential conversions). Propane cracking rate constants were determined at 475 °C through linear regressions.*

S.4. Textural characterization of commercial MFI, BEA, FAU and CHA zeolites

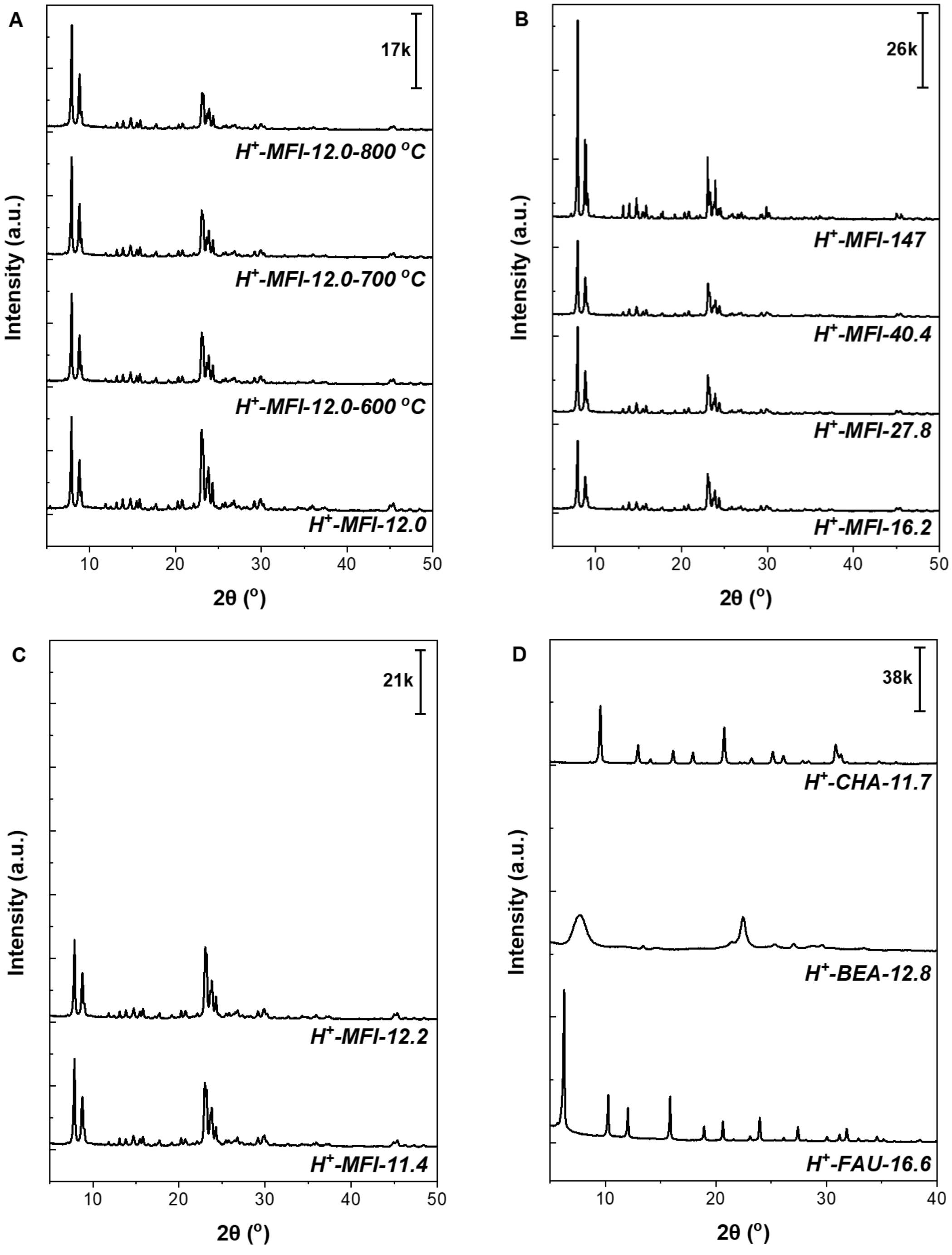

***Figure S7**: Powder XRD patterns of a) H^+-MFI-12.0, H^+-MFI-12.0-600 °C, H^+-MFI-12.0-700 °C and H^+-MFI-12.0-800 °C, b) H^+-MFI-16.2, H^+-MFI-27.8, H^+-MFI-40.4 and H^+-MFI-147, (c) H^+-MFI-11.4 and H^+-MFI-12.2, and (d) H^+-FAU-16.6, H^+-BEA-12.8 and H^+-CHA-11.7.*

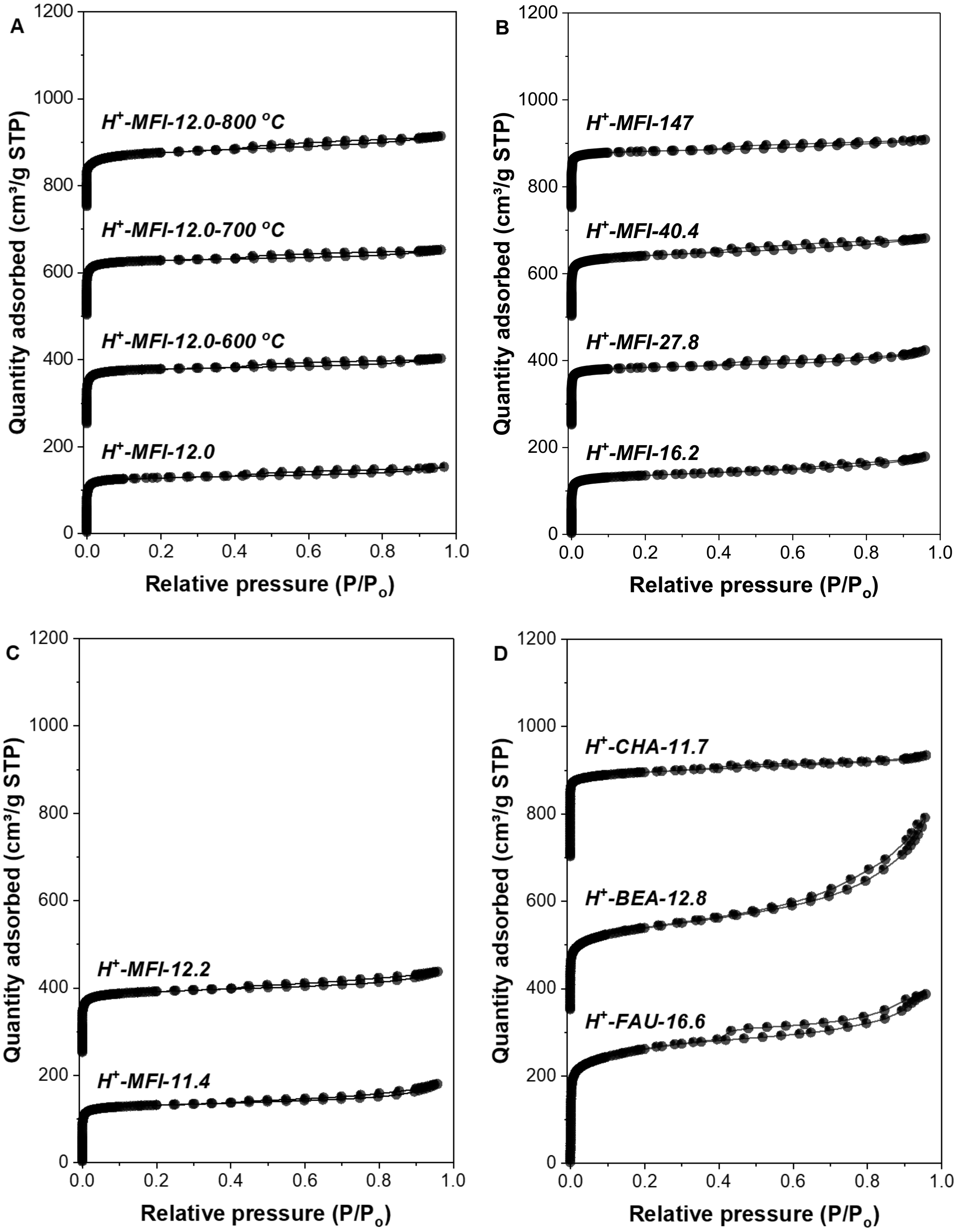

***Figure S8**: Ar adsorption isotherm performed at -186 °C (87.5 K) of a) H^+-MFI-12.0, H^+-MFI-12.0-600 °C, H^+-MFI-12.0-700 °C and H^+-MFI-12.0-800 °C, b) H^+-MFI-16.2, H^+-MFI-27.8, H^+-MFI-40.4 and H^+-MFI-147, (c) H^+-MFI-11.4 and H^+-MFI-12.2, and (d) H^+-FAU-16.6, H^+-BEA-12.8 and H^+-CHA-11.7. Isotherms are offset by 250 and 350 cm^3/g STP for (a-c) and (d), respectively.*

S.5. Supporting site quantification measurements

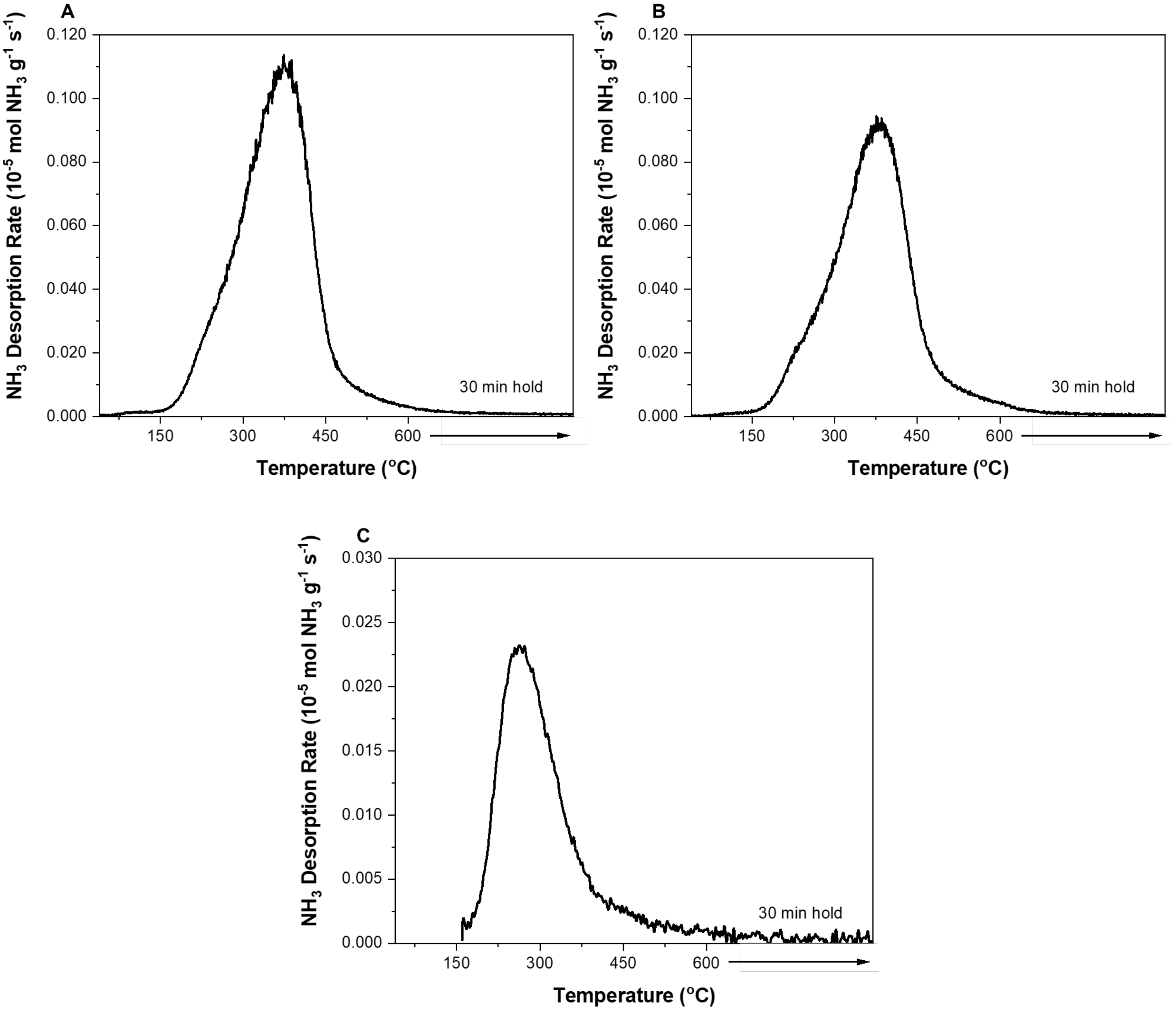

Figure S9: *Representative NH_3 temperature programmed desorption (TPD) profiles of (a) NH_4^+-MFI-11.4, (b) H^+-MFI-11.4 and (c) γ-Al_2O_3. TPD experiments were performed after Ar treatment at 500 °C for 1h. NH_3 wet purge at 40 °C was used for (a) & (b) and NH_3 dry purge at 160 °C for (c).*

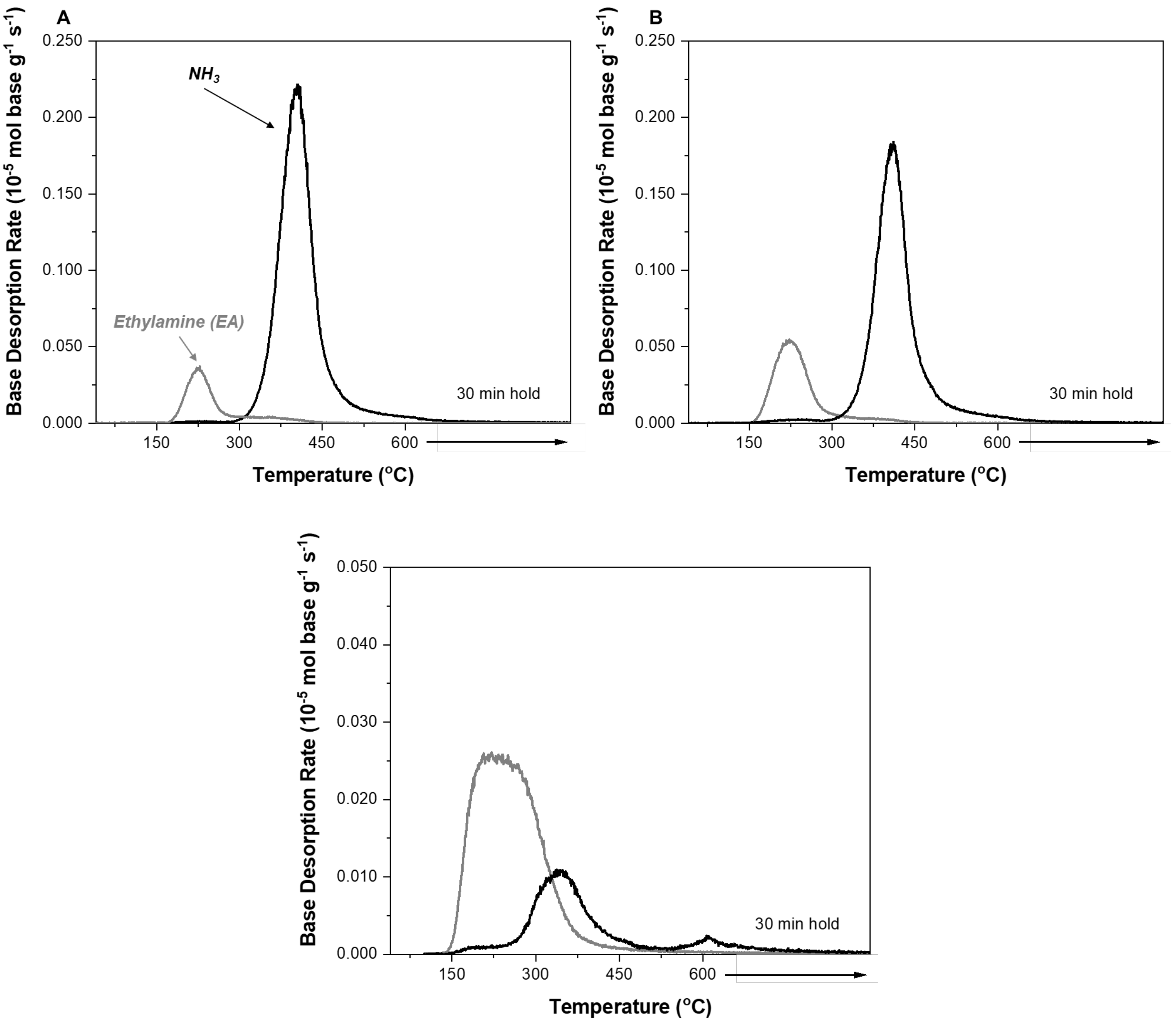

***Figure S10**: Representative Ethylamine (EA) temperature programmed desorption (TPD) profiles of (a) NH_4^+-MFI-11.4, (b) H^+-MFI-11.4 and (c) γ-Al_2O_3. TPD experiments were performed after Ar treatment at 500 °C for 1h. EA wet purge at 40 °C was used for (a) & (b) and EA dry purge at 100 °C for (c).*

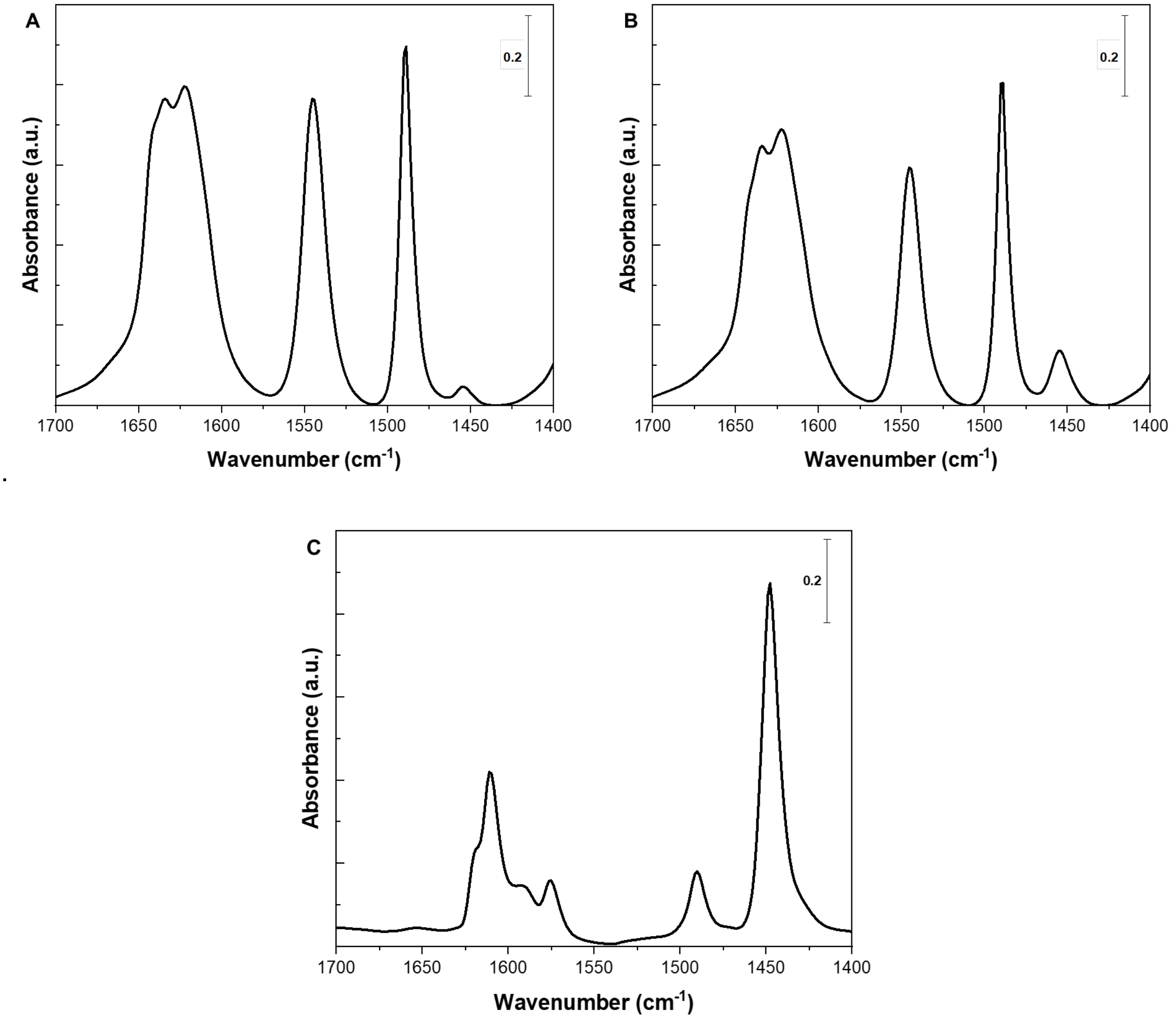

Figure S11: *Representative Pyridine (Py) transmission IR spectra of (a) NH_4^+-MFI-11.4, (b) H^+-MFI-11.4 and (c) γ-Al_2O_3. IR experiments were performed after Ar treatment at 500 °C for 1h. Py titration Brønsted & Lewis acid site counts were determined at 150 °C under vacuum.*

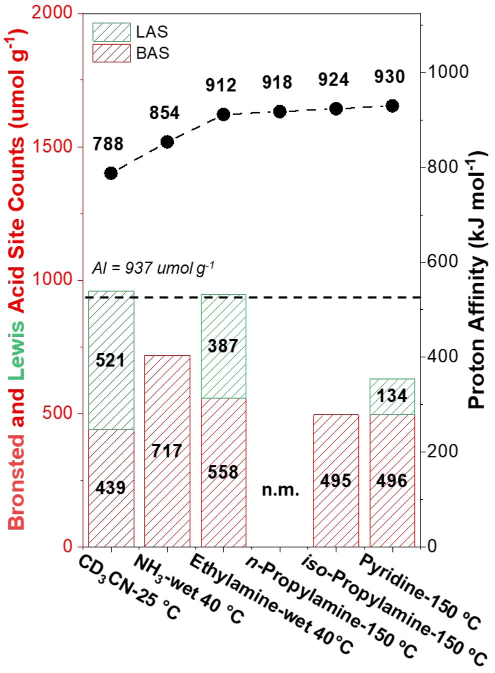

Figure S12: *Brønsted & Lewis acid site counts determined with transmission IR and temperature programmed desorption (TPD) experiments of H^+-BEA-12.8 with bases of varying proton affinity (obtained from NIST database) and size. Transmission IR spectra were collected after in-situ thermal dry air flow treatment at 500 °C for 1h of H^+-BEA-12.8 to determine pyridine (150 °C) and CD_3CN (25 °C) titration counts under vacuum. TPD experiments were performed after Ar treatment at 500 °C for 1h for NH_3, ethylamine and iso-propylamine with their denoted dry or wet purge temperature. Site count errors are within ≤ 5%.*

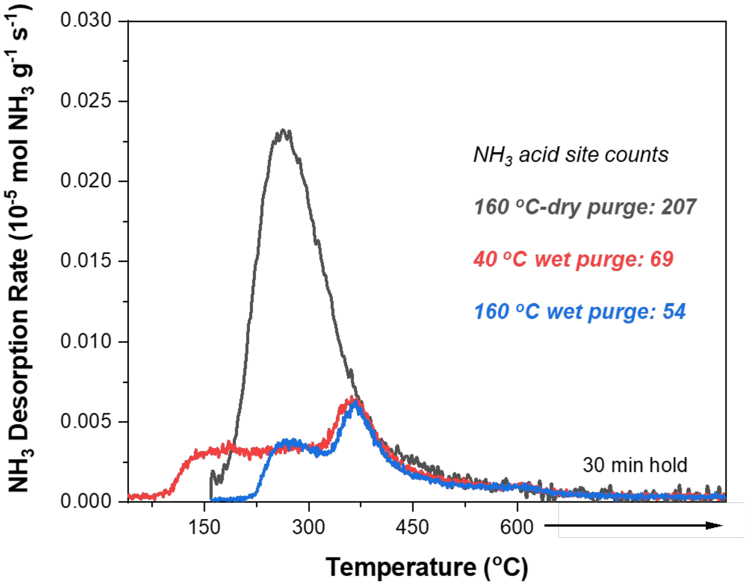

Figure S13: *NH_3 temperature programmed desorption (TPD) profile of γ-Al_2O_3 with varying purge protocol. TPD experiments were performed after Ar treatment at 500 °C for 1h for NH_3 with their denoted dry or wet purge temperature. Site count errors are within ≤ 5%.*

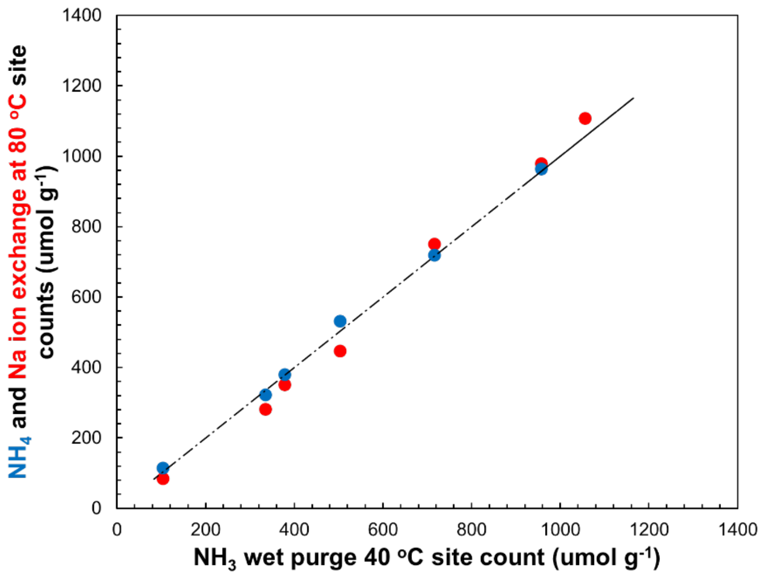

Figure S14: *Parity plot of Brønsted acid site (BAS) counts determined by NH_4^+ and Na^+ ion exchange at 80 °C vs determined by NH_3 wet purge at 40 °C for (a) commercial NH_4^+-MFI-12.0 and H^+-MFIs of varying Si/Al. Na^+ and BAS counts were determined with metal content analysis (ICP-OES) and temperature programmed desorption (TPD) experiments. TPD experiments were performed after Ar treatment at 500 °C for 1h for NH_3 wet purge and after He equilibration at 40 °C for 0.5 h for NH_4^+-form samples. Site count errors are within ≤ 5%.*

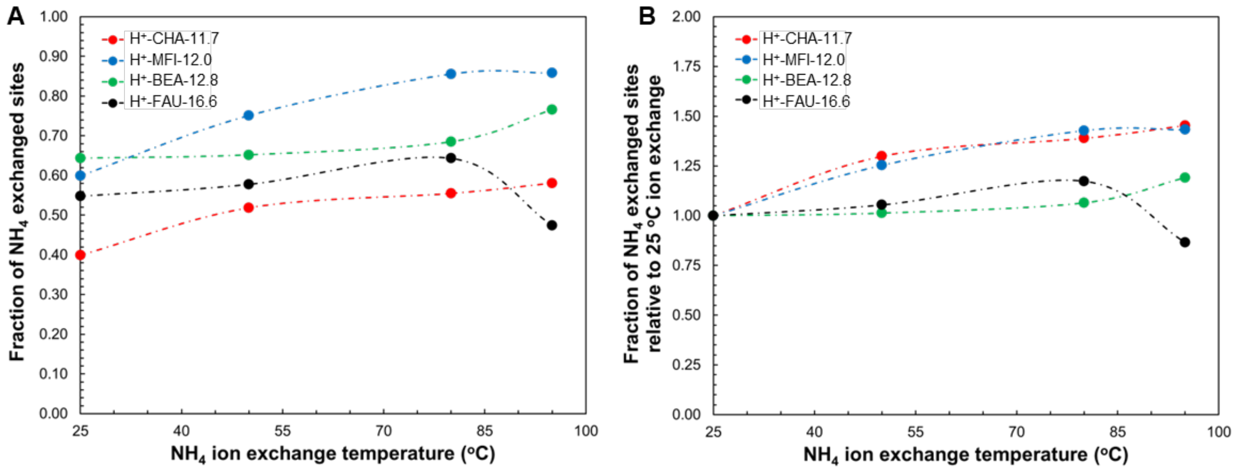

Figure S15: *(a) Fraction and (b) relative fraction of NH_4^+ exchanged sites versus ion exchange temperature for H^+-CHA-11.7, H^+-MFI-12.0, H^+-BEA-12.8 and H^+-FAU-16.6. TPD experiments were performed after He equilibration at 40 °C for 0.5 h for NH_4^+-form samples. Site count errors are within ≤ 5%.*

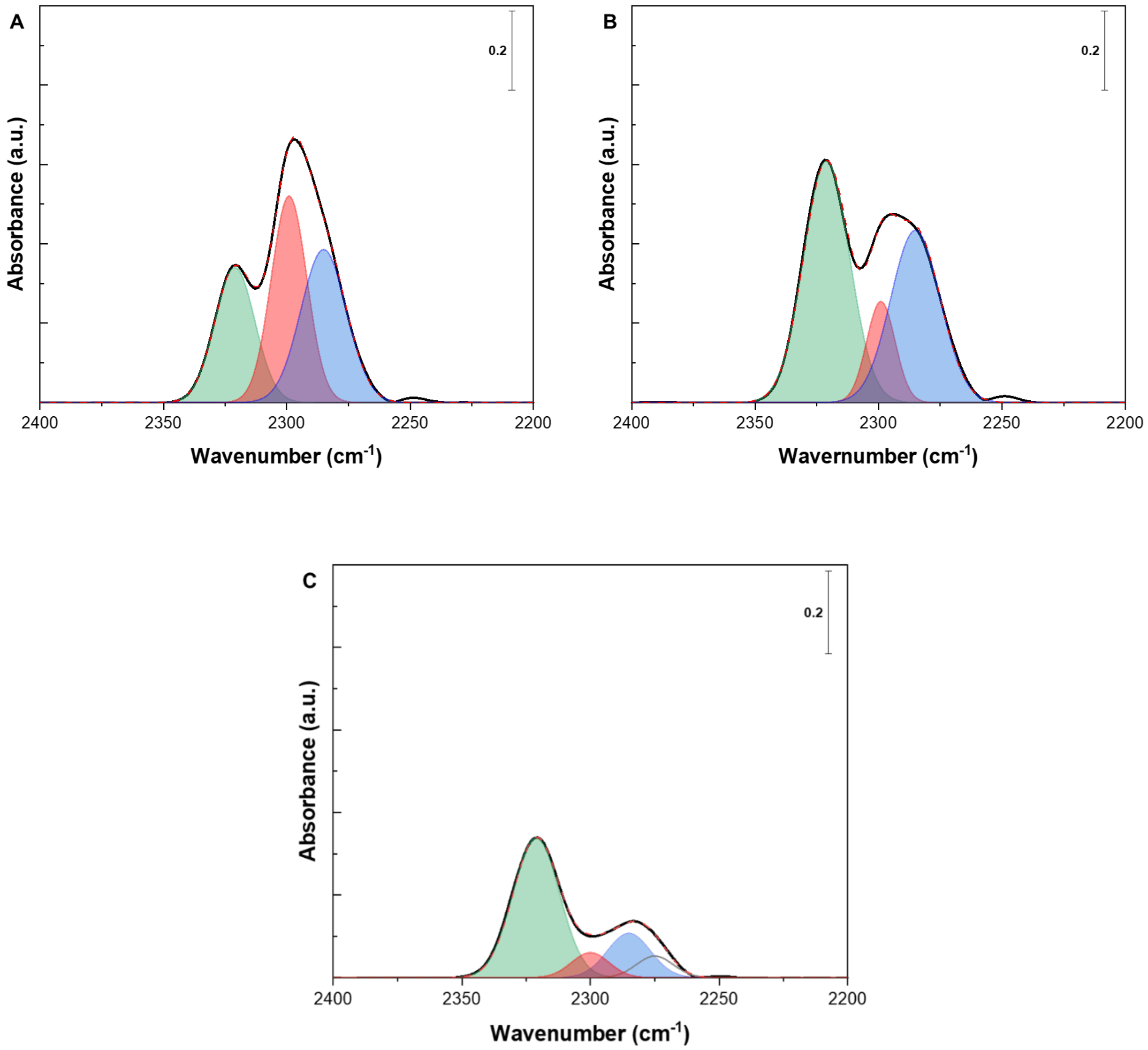

Figure S16*: Saturated CD_3CN transmission IR difference spectra of (a) H^+-MFI-12.0-600 °C, (b) H^+-MFI-12.0-700 °C and (c) H^+-MFI-12.0-800 °C at room temperature (25 °C). Transmission IR spectra were collected after in-situ thermal dry air flow treatment at 500 °C to determine CD_3CN (25 °C) titration counts under vacuum. In general, saturated CD_3CN difference IR spectra were deconvoluted into partially hydrolyzed sites (2330 - 2310 cm^{-1}), framework SiOHAl (2300 - 2297 cm^{-1}), Al-OH groups (2285 cm^{-1}) and Si-OH groups (2275 cm^{-1}) after 1h desorption.*
